# Two-Part and Related Regression Models for Longitudinal Data

**DOI:** 10.1146/annurev-statistics-060116-054131

**Published:** 2017-03

**Authors:** V.T. Farewell, D.L. Long, B.D.M. Tom, S. Yiu, L. Su

**Affiliations:** 1 Medical Research Council Biostatistics Unit, Institute of Public Health, University of Cambridge, Cambridge CB2 0SR, United Kingdom; 2 Department of Biostatistics, West Virginia University, Morgantown, West Virginia 26506

**Keywords:** longitudinal data, marginal covariate effects, mixture distributions, random effects, two-part models

## Abstract

Statistical models that involve a two-part mixture distribution are applicable in a variety of situations. Frequently, the two parts are a model for the binary response variable and a model for the outcome variable that is conditioned on the binary response. Two common examples are zero-inflated or hurdle models for count data and two-part models for semicontinuous data. Recently, there has been particular interest in the use of these models for the analysis of repeated measures of an outcome variable over time. The aim of this review is to consider motivations for the use of such models in this context and to highlight the central issues that arise with their use. We examine two-part models for semicontinuous and zero-heavy count data, and we also consider models for count data with a two-part random effects distribution.

## 1 Introduction

Statistical analysis based on two-part models arises in a variety of contexts. A simple, but common and useful, version of such models involves a model for a binary indicator variable and a model for another response variable given that the binary indicator takes one of the indicator’s two values. In this article we focus on this specific type of two-part models, as well as models with a comparable two-part structure for a random effects distribution in longitudinal settings.

An early technical discussion of this type of two-part model was given by Aitchison (1955) for modeling a nonnegative variable with a probability mass at zero and a continuous distribution for values greater than zero. It is common now to refer to such data as semicontinuous.

Another variant of this two-part model structure is often used for the analysis of zero-heavy count data. The structure was introduced by Cohen (1963) and given by Johnson & Kotz (1969), but was particularly popularized by Lambert (1992), who provided an excellent introduction with regression formulations. These models, the so-called zero-inflated Poisson (ZIP) models and their variants, combine a Poisson (or other distributions for count data) variable with a binary indicator variable for outcome, taking the value zero to accommodate the excess zeros that cannot be captured by the Poisson distribution. The departure from the models mentioned earlier is that the Poisson distribution also includes a probability mass for a zero observation, hence the model for the binary indicator variable is seen as inflating the probability of zero relative to the Poisson. In contrast, hurdle models (Cragg 1971) for counts or other types of data have a Bernoulli distribution for all the zero values and a separate distribution for nonzero observations. For count data, one of these is the zero-altered Poisson model, which differs from the ZIP model by having a Bernoulli distribution for a zero observation, and a truncated Poisson distribution with no probability mass assigned to zero for nonzero observations.

In the context of survival data, two-part models have been considered to capture the possibility of a cured fraction of patients, or long-term survivors, existing as a separate population. Early work was done by Boag (1949) and Berkson & Gage (1952). Subsequent work focused on regression settings (e.g., Farewell 1977, 1986) and more general time-to-event models, including semiparametric approaches (e.g., Taylor 1995). More information can be found in the book by Maller & Zhou (1996) and a recent review by Taweab & Ibrahim (2014). We do not deal with this particular application of two-part models. However, it is important to note that the plausibility of separate populations is essential to the use of two-part models in this context and is often important in other applications of such models.

There is a very large literature on the various types of two-part models with a correspondingly large number of areas of applications. These include machine failures, sexual behavior, nutrition, fertility, ecology, manufacturing, agriculture, and various economic datasets, including health care costs. This article does not aim to survey this literature. Our focus will be on the particular application of these two-part models in longitudinal settings when there are repeated measures over time from the same subject. With such longitudinal data, adapting these two-part models to account for within-subject correlation raises particular issues. In addition, other issues with two-part models, such as the interpretation of regression coefficients, may be even more problematic in the longitudinal settings. We address these issues and some approaches to dealing with them, predominately in the context of specific two-part models described in this article, but also for other similar models.

We primarily focus on likelihood-based approaches to two-part models with random effects for longitudinal semicontinuous (Sections 3, 4) and zero-heavy count (Section 5) data, and we also discuss counting process models with a two-part structure in the random effects distributions (Section 6). After describing the formulation and estimation of these likelihood-based models, we briefly comment on Bayesian and generalized estimating equations (GEE) approaches to the use of two-part models (Section 7). Then some important issues in the use of two-part models in longitudinal settings are highlighted and discussed (Section 8), and two primary examples from studies on psoriatic arthritis (PsA) (Sections 9, 10) and risky sexual behavior among HIV-positive individuals (Section 10) are presented to illustrate the use of the two-part models with particular emphasis on the issues raised.

## 2 Illustrative Examples

### 2.1 Quality of Life in Patients with Psoriatic Arthritis

PsA is a chronic inflammatory arthritis associated with psoriasis. The University of Toronto Psoriatic Arthritis (PsA) Clinic has been developing a prospective longitudinal observational cohort of patients with PsA since 1978 (Gladman et al. 1987). In a 2007 study, investigators were interested in examining whether there were differential effects of disease activity and damage on physical functioning as measured by the Health Assessment Questionnaire (HAQ) over the duration of PsA (Husted et al. 2007). In addition, there was a particular interest in genetics and the role of alleles that code for human leukocyte antigens (HLA) on disease progression and physical functioning in PsA patients (Gladman et al. 1998, Su et al. 2015).

The HAQ is a widely used self-reported functional status (disability) measure (Bruce & Fries 2003). The HAQ assesses physical function over the previous week and consists of 20 questions that cover 8 categories of daily living (i.e., dressing and grooming, arising, eating, walking, hygiene, reach, grip, and activities, including errands and chores). Patients rate their ability to perform a particular task within a category on a scale from 0 (no difficulty) to 3 (unable to do), with the highest score for any task within a category determining the score for that category. The scores for all 8 categories are then averaged to obtain an overall score on a scale from 0 (no disability) to 3 (severe disability) (Husted et al. 2005, 2007). Although discrete in nature (i.e., scores range from 0 to 3 in steps of 0.125), the overall HAQ score is generally treated as continuous when analyzed.

**Figure 1:**
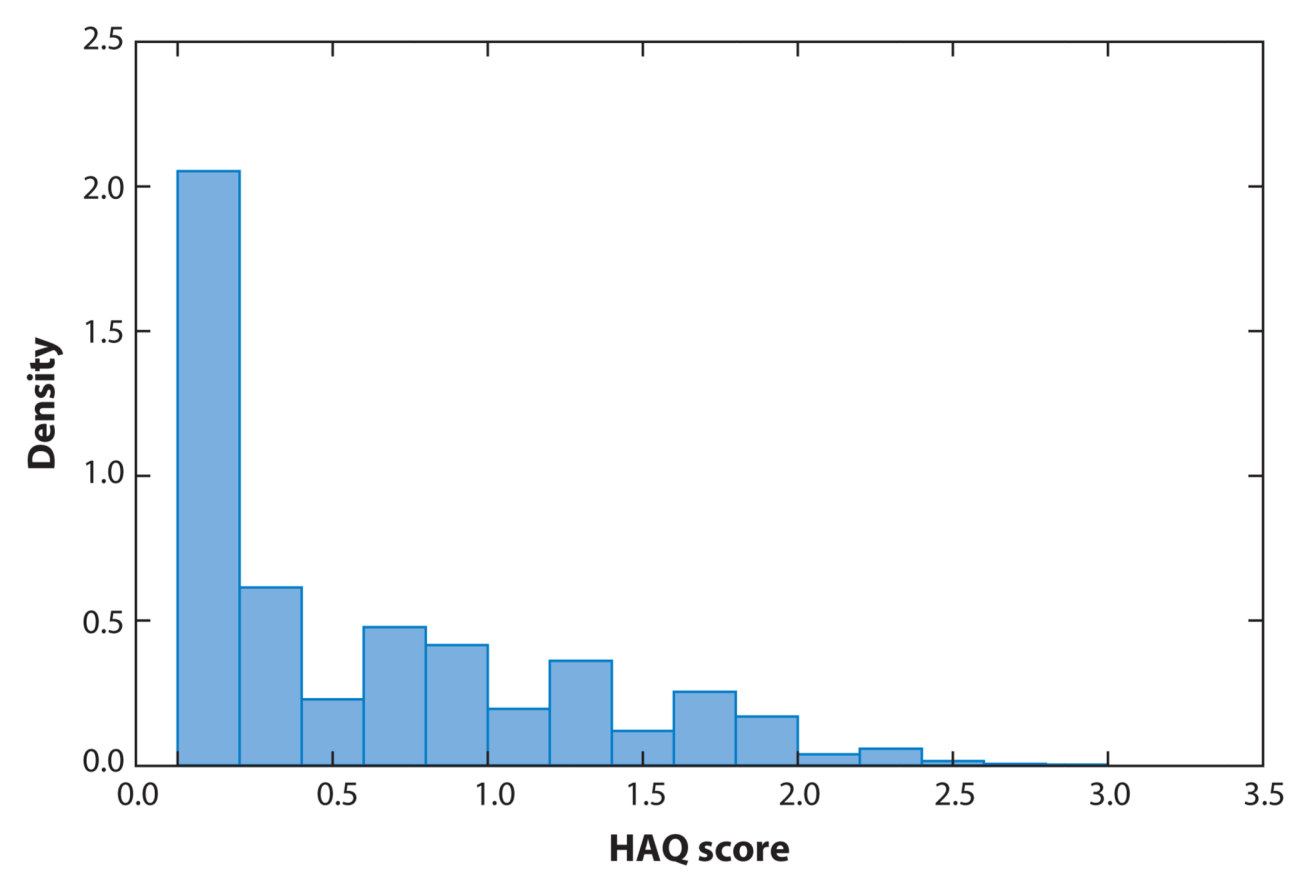
Bar plot of Health Assessment Questionnaire (HAQ) data from 382 psoriatic arthritis patients with 2,107 clinic visits. Adapted with permission from Su et al. (2009).

Since June 1993, the HAQ has been administered annually to patients in the PsA Clinic, and as of March 2005, 440 patients had completed at least one HAQ, with 382 (87%) completing two HAQs (Husted et al. 2007) and comprising the study group. In addition, at clinic visits, scheduled at 6–12 month intervals, demographic and other clinical information was obtained. There were 2,107 HAQ observations available for our analyses. Figure 1 illustrates clearly the cluster of 30.6% (645/2107) of the observations at zero.

### 2.2 Permanent Joint Damage in Patients with Psoriatic Arthritis

Another question of interest regarding PsA relates to what influences the development of permanent joint damage (defined as ankylosis, subluxation, or >20% decreased range of motion, attributable to joint damage rather than inflammation) (Siannis et al. 2006), which is often taken to be a measure of disease progression. However, a significant fraction of PsA patients, even with extended follow-up, are not observed to experience any damage in their joints. In the analysis of such data, a clinical question of interest is whether there exists a subpopulation of patients who will never experience damage. The existence of such a population will lead to an excess of zero observations because between any two longitudinal observation times, the patients in this subpopulation will always be observed to have no increase in their damaged joint count. This contrasts with the HAQ data, where there is an excess of zeros at each longitudinal observation time but the patients contributing to the excess may vary over time.

### 2.3 Motivational Interviewing Intervention on Risky Sexual Behaviors in HIV-Positive Patients

Reducing risky sexual behavior among people living with HIV/AIDS is one area of focus among infectious disease researchers. One measure of risky behavior is the number of unprotected anal or vaginal sexual intercourse acts (the UAVI count) within a given time period. The SafeTalk program was developed as a motivational interviewing-based intervention to reduce risky sexual behavior, particularly UAVI (Golin et al. 2010, 2012). To assess SafeTalk’s efficacy at reducing unprotected sex acts in this population, a randomized clinical trial was performed with subjects recruited at three sites being randomized to receive either SafeTalk or a nutritional intervention as control. The participants were then surveyed every four months for one year to measure their self-reported sexual acts in the previous three-month period. The research question for this study was whether those in the SafeTalk intervention had lower UAVI than those in the control group.

**Figure 2:**
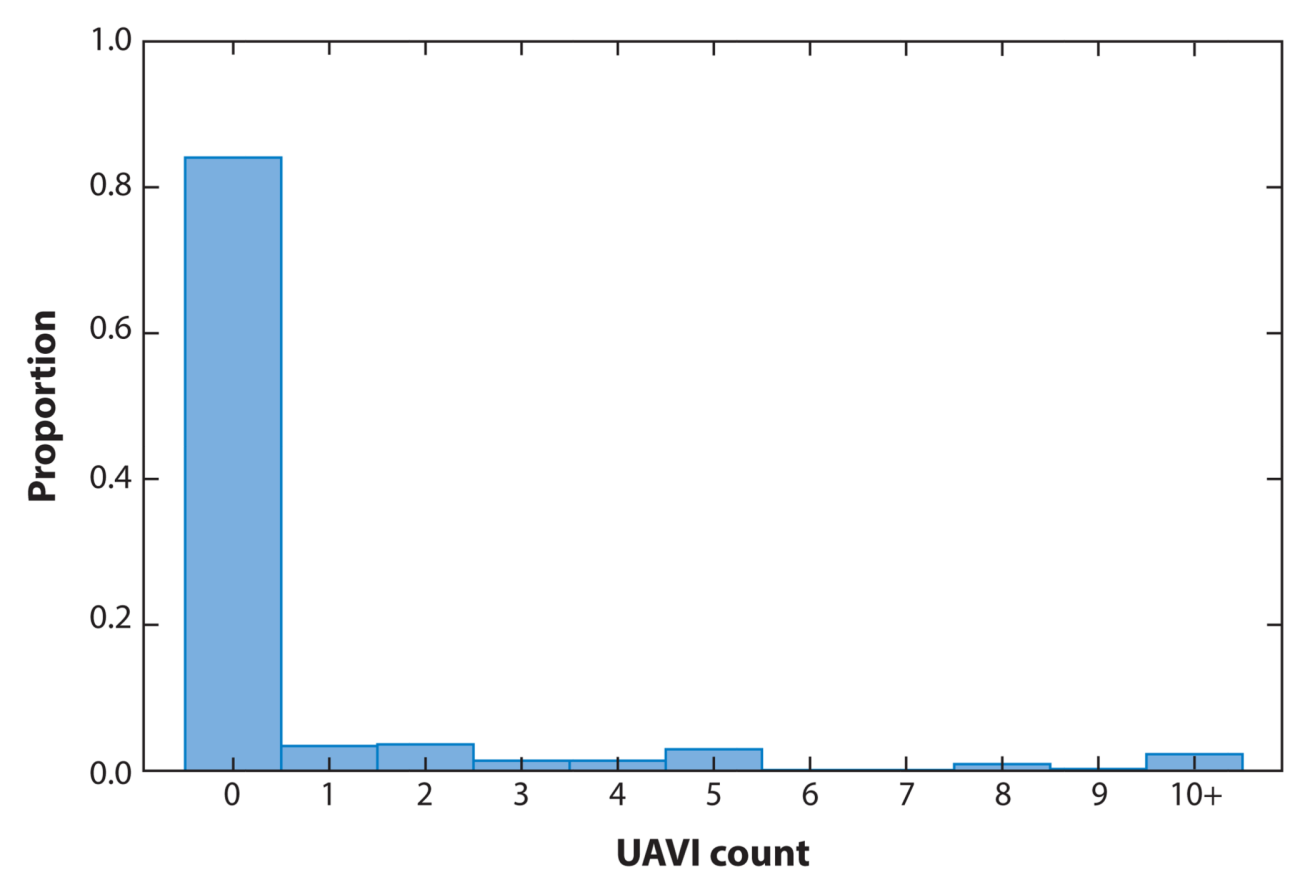
Bar plot of unprotected anal or vaginal sexual intercourse act (UAVI) count from 357 participants in the SafeTalk efficacy trial. Adapted with permission from Long et al. (2014).

For illustration, data from the 8-month follow-up for 357 participants with complete UAVI counts, excluding eight participants with UAVI counts greater than 18, are given in Figure 2. The data contain 300 (84%) zeros and 8 counts of 10+ (2.2%).

## 3 Two-Part Mixed Models for Longitudinal Semicontinuous Data

Semicontinuous data can be treated as a mixture of true zeros and continuously distributed positive values, which can be naturally viewed as generated from two processes, one determining whether the outcome is zero and the other determining the actual value if it is nonzero. For convenience, we refer to the data arising from these two processes as the binary part and the continuous part of the data, respectively.

Olsen & Schafer (2001) first extended the two-part model for application to longitudinal semicontinuous data by introducing correlated random effects into models for both the binary part and the continuous part. Tooze et al. (2002) discussed a similar two-part mixed model, and a general introduction was provided by Lachenbruch (2002). Here we follow the notation in Su et al. (2009) and describe the formulation for two-part mixed models briefly.

### 3.1 Model Formulation

Let *Y_ij_* be a semicontinuous variable for the *i*th (*i* = 1,…, *N*) subject at time *t_ij_* (*j* = 1,…, *n_i_*). This outcome variable can be represented by two variables, the occurrence variable

$$Z_{ij}=\{\begin{array}{ll} 0 & \text{if}\;Y_{ij}=0\\ 1 & \text{if}\;Y_{ij}>0 \end{array}$$

and the intensity variable *g*(*Y_ij_*) given that *Y_ij_* > 0, where *g*(⋅) is a transformation function (e.g., log) that makes *Y_ij_* | *Y_ij_* > 0 approximately normally distributed with a subject-time-specific mean and constant variance.

Instead of focusing on the marginal distribution of *Y_ij_*, in a two-part mixed model we are interested in both the distribution for the occurrence variable *Z_ij_* and the conditional distribution of the intensity variable *g*(*Y_ij_*) given that *Y_ij_* > 0. Specifically, it is assumed that *Z_ij_* follows a random effects logistic regression model

(1) $$\text{logit}\left\{\text{Pr}\left(Z_{ij}=1\;|\;{\text{X}}_{ij},U_{i}\right)\right\}={\text{X}}_{ij}\theta +U_{i},$$

where **X**_*ij*_ is a 1 × *q* covariate (used as a synonym for explanatory variable) vector, ***θ*** is a *q* × 1 regression coefficient vector, and *U_i_* is the subject-level random intercept. The intensity variable *g*(*Y_ij_*) given *Y_ij_* > 0 follows a linear mixed model

(2) $$g\left(Y_{ij}\right)\;|\;Y_{ij}>0,{\text{X}}_{ij}^{*}={\text{X}}_{ij}^{*}\beta +V_{i}+\epsilon_{ij},$$

where ${\text{X}}_{ij}^{*}$ is a 1 × *p* covariate vector, ***β*** is a *p* × 1 regression coefficient vector, and *V_i_* is again a subject-level random intercept. The error term *ϵ_ij_* is assumed to be normally distributed as $\text{N}(0,\sigma_{e}^{2}).$ Note that this two-part mixed model can be extended to include additional random effects. For illustration purposes and simplicity, we restrict attention here to two-part mixed models with random intercepts; extensions to models with random slopes are straightforward.

An important assumption is that the random intercepts, (*U_i_*, *V_i_*), are jointly normal and possibly correlated,

(3) $$\left[\begin{array}{c} U_{i}\\ V_{i} \end{array}\right]∼\text{N}\left(\left[\begin{array}{c} 0\\ 0 \end{array}\right],\left[\begin{array}{cc} \sigma_{u}^{2} & \rho \sigma_{u}\sigma_{v}\\ \rho \sigma_{u}\sigma_{v} & \sigma_{v}^{2} \end{array}\right]\right).$$

In the context of the HAQ example, the correlation aspect of this assumption can be interpreted as follows: The presence or absence of disability at one time point is related to the level of disability, if any, at that and other time points.

In this model, the covariate vectors **X**_*ij*_, ${\text{X}}_{ij}^{*}$ may coincide, but this is not required. The data can be unbalanced by design or because of ignorable missingness. The primary targets of inference are the regression coefficients ***θ*** and ***β***, and variance components, including the correlation parameter *ρ*, are usually treated as nuisance parameters.

### 3.2 Model Estimation

The estimation of ***θ***, ***β***, $\sigma_{u}^{2},$ $\sigma_{v}^{2},$ *ρ* and $\sigma_{e}^{2}$ can be based on maximization of the likelihood

(4) $$\begin{array}{l} L=\prod_{i=1}^{N}\int_{u_{i}}\int_{v_{i}}\prod_{j=1}^{n_{i}}f\left(y_{ij}\;|\;\text{θ},\text{β},u_{i},v_{i},\sigma_{e}^{2}\right)f\left(u_{i},v_{i}\;|\;\sigma_{u}^{2},\sigma_{v}^{2},\rho \right)\text{d}v_{i}\text{d}u_{i}\\ \;=\prod_{i=1}^{N}\int_{u_{i}}\int_{v_{i}}\prod_{j=1}^{n_{i}}{\left\{1-\mathrm{Pr}\left(Z_{ij}=1\;|\;\text{θ},u_{i}\right)\right\}}^{\left(1-z_{ij}\right)}{\left\{\mathrm{Pr}\left(Z_{ij}=1\right)\;|\;\text{θ},u_{i}\right\}}^{z_{ij}}\\ \;\times {\left[f\left\{g\left(y_{ij}\right)\;|\;\text{β},v_{i},\sigma_{e}^{2}\right\}\right]}^{Z_{ij}}f\left(u_{i},v_{i}\;|\;\sigma_{u}^{2},\sigma_{v}^{2},\rho \right)\text{d}v_{i}\text{d}u_{i}, \end{array}$$

which presents the same computational challenges as with generalized linear mixed models (GLMMs) (Stiratelli et al. 1984, Breslow & Clayton 1993, Wolfinger & O’Connell 1993) owing to the fact that the likelihood cannot be evaluated exactly because of the intractable integrals. Olsen & Schafer (2001) proposed an approximate Fisher scoring procedure based on high-order Laplace approximations for obtaining maximum likelihood estimates. Tooze et al. (2002) used quasi-Newton optimization of the likelihood approximated by adaptive Gaussian quadrature and implemented it in the SAS PROC NLMIXED procedure (SAS Institute Inc. 2013).

Although there has been some discussion of the ability to recover the true parameter values and the computational efficiency of different methods for handling the intractable integrals encountered in GLMMs, less work has been done with regard to what properties of the data (e.g., mean cluster size, proportion of zeros, intraclass correlation, etc.) and model lead to issues with estimation. In Su et al. (2009), some investigation was made into instability of estimation for parameters in the binary part of two-part models. They found that when the unexplained between-subject variability in the binary part was large or the proportion of zeros was small, this could lead to instability, owing to the (profile) likelihood surface being flat. Thus it is important to avoid omitting important explanatory variables when specifying the regression structure of the binary part, as such omission may lead to unstable estimation of variance components and subject-specific coefficients. Further investigations are needed to determine what aspects of the data and model may lead to estimation issues.

## 4 An Alternative Two-Part Model for Longitudinal Semicontinuous Data

The two-part mixed model described in Section 3.1 examines subject-specific (conditional) effects of covariates on the two processes of the semicontinuous outcome. In certain scenarios, the population-averaged (marginal) effects of covariates are more desirable. For example, it is of interest to examine the population-averaged effects of prespecified genetic markers on physical functioning in the PsA cohort.

Su et al. (2015) developed an alternative two-part mixed model where the population-averaged effects of covariates for the binary part of the model are directly parameterized. This model can be conveniently implemented using standard software procedures such as SAS NLMIXED. Also, compared with the moment-based approaches in Hall & Zhang (2004), it can deal with longitudinal data that is unbalanced either by design or owing to ignorable missingness (such as so-called missing at random data) because it is fully likelihood-based (Heagerty 2002, Diggle et al. 2002). In addition, it can offer some degree of robustness in regression parameter estimation for the binary part of the model for departures from the true underlying random effect structure. Here we briefly describe this alternative two-part model for longitudinal semicontinuous data.

### 4.1 Model Formulation

Basically, Su et al. (2015) replaced the random effect *U_i_* in Equation 1 by a random effect *B_i_* that is assumed to follow the bridge density of Wang & Louis (2003):

$$f_{B}\left(b_{i}\;|\;\phi \right)=\frac{1}{2\pi }\frac{\sin\left(\phi \pi \right)}{\text{cosh}\left(\phi b_{i}\right)+\cos\left(\phi \pi \right)}\;\left(-\infty <b_{i}<\infty \right),$$

with unknown parameter *ϕ* (0 < *ϕ* < 1). This bridge distribution is symmetric with mean zero and variance $\sigma_{b}^{2}=\pi^{2}\left(\phi^{-2}-1\right)/3.$ It is slightly heavy tailed and more concentrated than the normal distribution with the same variance. The key characteristic of this bridge density is that after integration over the random intercepts in the two parts, (*B_i_*, *V_i_*), with *V_i_* in Equation 2 normally distributed as before, the marginal probability Pr(*Z_ij_* = 1) relates to the linear predictors through the same logit link function as for the corresponding conditional probability. In addition, if we specify the marginal regression structure of the binary part as

$$\text{logit}\left\{\text{Pr}\left(Z_{ij}=1\;|\;{\text{X}}_{ij}\right)\right\}={\text{X}}_{ij}\theta ,$$

then the marginal effects of covariates ***θ*** are proportional to the subject-specific conditional effects of covariates $\tilde{\theta },$ with $\theta =\phi \tilde{\theta }.$ Therefore, we could replace Equation 1 as

$$\text{logit}\left\{\text{Pr}\left(Z_{ij}=1\;|\;{\text{X}}_{ij},B_{i}\right)\right\}={\text{X}}_{ij}\theta /\phi +B_{i}.$$

Based on marginalization of random effects models, Heagerty (1999) and Heagerty & Zeger (2000) proposed full likelihood-based methods of estimating marginal regression parameters for longitudinal binary data. In their models, random effects are assumed to be normally distributed and the marginal probability and the conditional probability given the random effects are matched by an intercept term Δ*_ij_*. Similarly, in this model we have

$$\text{Pr}\left(Z_{ij}=1\;|\;{\text{X}}_{ij}\right)=\int \text{Pr}\left(Z_{ij}=1\;|\;b_{i}\right)f_{B}\left(b_{i}\right)\text{d}b_{i}=\int \text{log}{\text{it}}^{-1}\left(\Delta_{ij}+b_{i}\right)f_{B}\left(b_{i}\right)\text{d}b_{i},$$

and the intercept term is actually $\Delta_{ij}={\text{X}}_{ij}\tilde{\theta }.$

For the model for the continuous part, we let *V_i_* be normally distributed with mean zero and variance $\sigma_{v}^{2}.$ Therefore, *g*(*Y_ij_*) | *Y_ij_* > 0 given the random intercepts (*B_i_*, *V_i_*) again follows a normal linear mixed model with mean ${\text{X}}_{ij}^{*}\beta +V_{i}$ and variance $\sigma_{e}^{2}.$

As in the model of Section 3.1, a relationship between the two processes that generate semicontinuous data should be allowed, especially if the outcome is observed at multiple time points. For this purpose, a bivariate joint distribution for the random intercepts (*B_i_*, *V_i_*) can be constructed from a pair of normal random variables

$$\left[\begin{array}{c} U_{i}\\ V_{i} \end{array}\right]∼\text{N}\left(\left[\begin{array}{c} 0\\ 0 \end{array}\right],\left[\begin{array}{cc} 1 & \rho \sigma_{v}\\ \rho \sigma_{v} & \sigma_{v}^{2} \end{array}\right]\right),$$

and the probability integral transformation

$$B_{i}=F_{B}^{-1}\left\{\Phi \left(U_{i}\right)\right\},\;\text{where}\;F_{B}^{-1}\left(x\right)=\frac{1}{\phi }\text{log}\left[\frac{\text{sin}\left(\phi \pi x\right)}{\text{sin}\left\{\phi \pi \left(1-x\right)\right\}}\right],0<x<1,$$

can be used to obtain *B_i_* (Wang & Louis 2003, Lin et al. 2010), where $F_{B}^{-1}\left(\cdot \right)$ is the inverse cumulative distribution function associated with the bridge density. Φ(·) is the cumulative distribution function of the standard Normal. Lin et al. (2010) found that the correlation for (*B_i_*, *V_i_*) is approximately the same as the correlation *ρ* for (*U_i_*, *V_i_*).

### 4.2 Model Estimation

In this two-part model, one primary target of inference is likely to be the marginal effects of covariates ***θ*** that are relevant to the model for the binary part. The regression coefficients in the model for the continuous part, ***β***, would also likely be of interest, whereas variance components $\sigma_{b}^{2}$ (or equivalently *ϕ*), $\sigma_{v}^{2},\sigma_{e}^{2},$ and the correlation parameter *ρ* will usually be regarded as nuisance parameters. The estimation of ***θ***, ***β***, $\sigma_{b}^{2},$ $\sigma_{v}^{2},$ *ρ* and $\sigma_{e}^{2}$ is based on maximization of the likelihood in Equation 4, but with *b_i_* and $\sigma_{b}^{2}$ replacing *u_i_* and $\sigma_{u}^{2},$ respectively.

## 5 Zero-Inflated Poisson Models with Random Effects for Longitudinal Count Data

As discussed in the Introduction, zero-inflated models for count data allow observed zeros to arise both from the binary part of the model and as an observation from a Poisson distribution or other distributions for count data. This is in contrast to the two-part model structure for semicontinuous data discussed in previous sections. Extending Lambert’s ZIP model (Lambert 1992), Hall (2000) proposed a ZIP model with random effects in the Poisson process to account for within-subject correlation. In order to account for overdispersion in addition to excess zeros in correlated data, Yau et al. (2003) proposed a zero-inflated negative binomial (ZINB) regression model with independent random effects in each process. In this section, we focus on such random effects models for zero-inflated count data, maintaining as much consistency in notation as possible with Sections 3.1 and 4.1.

### 5.1 Model Formulation

Let*Y_ij_* be the a count variable for the *i*th (*i* = 1,…, *N*) subject at time *t_ij_* (*j* = 1,…, *n_i_*). It is linked to a binary variable *Z_ij_* such that:

$$Y_{ij}∼\{\begin{array}{ll} 0 & \text{with}\;\text{probability}\;\text{Pr}\left(Z_{ij}=0\;|\;{\text{X}}_{ij},U_{i}\right)\\ \text{Poisson}\left(\mu_{ij}^{C}\right) & \text{with}\;\text{probability}\;\text{Pr}\left(Z_{ij}=1\;|\;{\text{X}}_{ij},U_{i}\right)=1-\text{Pr}\left(Z_{ij}=0\;|\;{\text{X}}_{ij},U_{i}\right), \end{array}$$

where $\mu_{ij}^{C}=E\left(Y_{ij}\;|\;Z_{ij}=1,{\text{X}}_{ij}^{*},V_{i}\right).$ As mentioned earlier, we have used similar notation to that used in Sections 3.1 and 4.1 for models for semicontinuous data. However, conceptually *Z_ij_* is here regarded as a partially latent, not a fully observed, variable. This is because if *Y_ij_* > 0, then *Z_ij_* = 1, but if *Y_ij_* = 0 then it is not known whether *Z_ij_* = 0 or *Z_ij_* = 1. The notation $\mu_{ij}^{C}$ indicates that the Poisson mean is conditional on the random effect *V_i_* and *Z_ij_* = 1, where *Z_ij_* follows a random effects logistic regression model

$$\text{logit}\{\text{Pr}(Z_{ij}=1\;|\;{\text{X}}_{ij},U_{i})\}={\text{X}}_{ij}\theta +U_{i},$$

as in Section 3.1, although covariate effects are now linked to the partially latent variable *Z_ij_*. The Poisson mean, $\mu_{ij}^{C},$ is modeled log-linearly as

(5) $$\text{log}(\mu_{ij}^{C})=X_{ij}^{*}\beta +V_{i},$$

with ${\text{X}}_{ij}^{*},$ ***β*** and *V_i_* defined similarly to in Section 4. Where appropriate, an offset, log(*O_ij_*), might be added to the right hand side of Equation 5. As in Section 3.1, for simplicity, we restrict attention here to ZIP models with random intercepts, although the models can easily be extended. Also, as in Section 3.1, the common assumption would be that the random intercepts, (*U_i_*, *V_i_*), are jointly normal and possibly correlated.

### 5.2 Model Estimation

The estimation of ***θ***, ***β***, $\sigma_{u}^{2},\sigma_{v}^{2},$ and *ρ* can be based on maximization of the likelihood

$$L=\prod_{i=1}^{N}\int_{u_{i}}\int_{v_{i}}\prod_{j=1}^{n_{i}}\text{Pr}\left(y_{ij}\;|\;\text{θ},\text{β},u_{i},v_{i}\right)f\left(u_{i},v_{i}\;|\;\sigma_{u}^{2},\sigma_{v}^{2},\rho \right)\text{d}v_{i}\text{d}u_{i},$$

where

$$\begin{array}{l} \text{Pr}\left(y_{ij}\;|\;\text{θ},\text{β},u_{i},v_{i}\right)={\left[\text{Pr}\left(Z_{ij}=0\;|\;{\text{X}}_{ij},u_{i}\right)+\left(1-\text{Pr}\left(Z_{ij}=0\;|\;{\text{X}}_{ij},u_{i}\right)\right)e^{-\mu_{ij}^{C}}\right]}^{1-z_{ij}}\\ \times {\left[\frac{\left(1-\text{Pr}\left(Z_{ij}=0\;|\;{\text{X}}_{ij},ui\right)\right)e^{-\mu_{ij}^{C}}{\left(\mu_{ij}^{C}\right)}^{y_{ij}}}{y_{ij}!}\right]}^{z_{ij}}. \end{array}$$

Using the expectation-maximization (EM) algorithm framework that Lambert (1992) proposed, Hall (2000) fitted this ZIP model with random effects with the EM algorithm using Gaussian quadrature. Although Hall (2000) only accounted for correlation within the Poisson process, others have utilized correlated random effects in both processes of the ZIP and hurdle models for longitudinal count data (Dobbie & Welsh 2001, Min & Agresti 2005, Ghosh & Albert 2009, Neelon et al. 2011, Lee et al. 2006). As mentioned in Section 1, hurdle models are an alternative to zero-inflated models and have been extended to longitudinal settings (Min & Agresti 2005). Similar to semicontinuous models, these two-part models address all zeros separately from the positive realizations, using Bernoulli and truncated count distributions, respectively. Rather than the partially latent class *Z_ij_* described for the zero-inflated model, the *Z_ij_* for hurdle models is equivalent to the *Z_ij_* from Section 3.1.

## 6 Models Based on Two-Part Random Effects Distributions for Longitudinal Count Data

Another two-part model structure for longitudinal count data that is appropriate in some contexts is to have a binary component to the model, which indicates if all longitudinal counts for a subject must be zero, and a second component for the distribution of the counts if this is not the case. Basically, in certain scenarios, it is believed that there exists a separate population (e.g., PsA patients without any joint damage over time) with all longitudinal observations equal to zero. The two-part model structure discussed in Section 5 could be adapted to deal with this scenario if the binary part of the model were altered to accommodate this specific case of having a positive probability of always having zeros for each subject.

However, an alternative approach is to achieve the same effect by adopting a two-part or similar model for a random effects distribution. Models of this type are sometimes termed mover-stayer models, and assume that there are two populations of subjects: stayers, who have no probability of a nonzero observation, and movers, who may have a nonzero observation at one or more time points. A version of this type of model for count data is outlined in this section. The presentation is based on that of Yiu et al. (2016), who used such a model for modeling joint damage in PsA patients, and this particular application is subsequently considered.

### 6.1 Patient-Level Random Effects Models

In this section, the general form of patient-level random effects models is given, followed by a description of the particular random effects distributions used subsequently.

#### 6.1.1 Model formulation

Let *Y_ij_* be a count variable for the *i*th (*i* = 1,…, *N*) subject at the *j*th visit time (*j* = 1,…, *n_i_*). Assume *Y_ij_* is Poisson distributed with mean

$$u_{i}\Lambda_{ij}=u_{i}O_{ij}\lambda_{0}\;\text{exp}\left({\text{X}}_{ij}\beta \right),$$

where *u_i_* is a realization of the patient-level random effect *U_i_*, which induces correlation between the observations of a patient; log(*O_ij_*) is an offset, typically introduced to allow for irregularly spaced observations; λ_0_ is a constant baseline intensity; and ***β*** and **X***_ij_* are row vectors of regression coefficients and covariates associated with the *j*th observation respectively.

To account for a subpopulation of stayers, the distribution of *U_i_* is taken to have a two-part distribution. Specifically the mover-stayer random effects densities for *U_i_* are of the form

$$g_{M-S}\left(u_{i}\right)=\{\begin{array}{ll} \pi_{i}, & \text{if}\;u_{i}=0\\ f\left(u_{i}\right), & \text{if}\;u_{i}>0, \end{array}$$

where *π_i_* is the probability that the *i*th patient is a stayer and *f*(*u_i_*) is a truncated random effect density which integrates to 1 − *π_i_* when the *i*th patient is a mover. The corresponding marginal likelihood contribution from the *i*th patient, *L_i_*, is then

$${\left\{\int_{0}^{\infty }\prod_{j=1}^{n_{i}}\frac{{\left(u_{i}\wedge_{ij}\right)}^{y_{ij}}\text{exp}\left(-u_{i}\wedge_{ij}\right)}{y_{ij}!}f\left(u_{i}\right)\text{d}u_{i}\right\}}^{c_{i*}}{\left\{\pi_{i}+\int_{0}^{\infty }\prod_{j=1}^{n_{i}}\text{exp}\left(-u_{i}\wedge_{ij}\right)f\left(u_{i}\right)\text{d}u_{i}\right\}}^{1-c_{i*}},$$

where *c*_i_*__ = 0 if the *i*th patient remained damage free while in the clinic and *c*_i_*__ = 1 otherwise. Models corresponding to a likelihood of this form can be referred to as Poisson M-S models, and further qualification, when needed, can be through the addition of the type of random effects distribution used.

#### 6.1.2 Random effects distributions

There can be various mover-stayer random effects densities chosen for *U_i_*. In Subsection 11.1 we consider the three different two-parameter random effects distributions used by O’Keeffe et al. (2012), which may capture the behavior of *U_i_* at and near zero differently. The first two random effects distributions are of the two-part form

$$g_{M-S}\left(u_{i}\right)=\{\begin{array}{ll} \pi_{i} & \text{if}\;u_{i}=0\\ \left(1-\pi_{i}\right)g\left(u_{i}\right) & \text{if}\;u_{i}>0, \end{array}$$

where *g*(*u_i_*) has either a gamma density with rate and shape parameter $\frac{1}{\theta }$ or an inverse Gaussian density with mean 1 and shape parameter *ψ*. That is,

$$g\left(u_{i}\;|\;\theta \right)=\frac{{\left(\frac{1}{\theta }\right)}^{\frac{1}{\theta }}}{\Gamma \left(\frac{1}{\theta }\right)}u_{i}^{\frac{1}{\theta }-1}\;\text{exp}\left(-\frac{u_{i}}{\theta }\right)\;\text{or}\;g\left(u_{i}\;|\;\psi \right)={\left(\frac{\psi }{2\pi u_{i}^{3}}\right)}^{\frac{1}{2}}\;\text{exp}\left(-\frac{{\psi \left(u_{i}-1\right)}^{2}}{2u_{i}}\right).$$

These two distributions will be referred to as the M-S gamma and M-S inverse Gaussian, respectively. The third mover-stayer random effects density is a compound Poisson (CP) of the form $U_{i}=\sum_{j=1}^{K_{i}}L_{j},$ where *K_i_* is a Poisson random variable with rate parameter *ρ_i_* and *L_j_* (*j* = 1,…, *K_i_*) are independently and identically distributed gamma random variables with shape and rate parameters 1 and *ν*, respectively. The density is then given by

$$g_{M-S}\left(u_{i}\;|\;v,\rho_{i}\right)=\text{exp}\left(-\rho_{i}-vu_{i}\right)\sqrt{\frac{v\rho_{i}}{u_{i}}}I_{1}\left(2\sqrt{v\rho_{i}u_{i}}\right),\;\text{where}\;I_{1}\left(h\right)=\sum_{k=0}^{\infty }\frac{1}{k!\Gamma \left(k+2\right)}{\left(\frac{h}{2}\right)}^{2k+1}$$

is a modified Bessel function of the first kind. The CP density contains a point mass exp(−*ρ_i_*) at zero and a density along the positive real line, and hence its density is conveniently in the mover-stayer form with *π_i_* = exp(−*ρ_i_*). Readers are directed to Aalen (1992) and Moger (2004, 2005) for applications of the CP distribution to survival studies.

Another commonly used random effects distribution for the positive part is the Log Normal. It can be shown, however, that this distribution is very similar to that of an inverse Gaussian.

### 6.2 Patient- and Observation-Level Random Effects Models

In some applications, it may be desirable to allow for time-varying unobserved heterogeneity in longitudinal count data. One way to introduce this into the Poisson M-S models is through the incorporation of observation-level random effects. In this extended model, the patient-level random effects primarily introduce correlation between observations within patients, and the observation-level random effects are introduced for capturing unobserved heterogeneity.

Let *U_i_* and *U_ij_* be multiplicative patient-level mover-stayer and observation-level random effects, respectively. Assume *Y_ij_* is Poisson distributed with mean

$$u_{i}u_{ij}\Lambda_{ij}=u_{i}u_{ij}O_{ij}\lambda_{0}\;\text{exp}\left({\text{X}}_{ij}\beta \right).$$

Then, under the usual assumption that *U_i_* and *U_ij_* are independent, the marginal likelihood contribution, *L_i_*, from the *i*th patient is

$${\left\{\int_{0}^{\infty }\prod_{j=1}^{n_{i}}h\left(y_{ij}\;|\;u_{i};\Lambda_{ij}\right)f\left(u_{i}\right)\text{d}u_{i}\right\}}^{c_{i_{*}}}{\left\{\pi_{i}+\int_{0}^{\infty }\prod_{j=1}^{n_{i}}h\left(0\;|\;u_{i};\Lambda_{ij}\right)f\left(u_{i}\right)\text{d}u_{i}\right\}}^{1-c_{i_{*}}},$$

where *c*_i_*__ is as before and

$$h\left(y\;|\;u_{i};\Lambda_{ij}\right)=\int_{0}^{\infty }\frac{{\left(u_{ij}u_{i}\Lambda_{ij}\right)}^{y}\;\text{exp}(-u_{ij}u_{i}\Lambda_{ij})}{y!}g\left(u_{ij}\right)\text{d}u_{ij}.$$

This model can be implemented with patient-level mover-stayer random effects distributions such as those in Section 6.1.2. The observation-level random effects distribution, *g*(*u_ij_*), follows a gamma distribution that takes the same form as given in Section 6.1.2 but with parameter *θ^nb^*. These models may be termed negative binomial M-S (NB M-S) models with further qualification by the type of patient-level random effects distribution used. Note that a ZINB model is obtained from the NB M-S gamma and NB M-S inverse Gaussian models when *θ* and $\frac{1}{\psi }=0,$ respectively. However, unlike the models in Section 5, the zero inflation is at the patient level and not the observation level. The class of Poisson M-S models is obtained when *θ^nb^* = 0.

### 6.3 Model Estimation

The estimation of λ_0_, ***β***, and the parameters contained in the random effects distributions can be based on maximization of the likelihood

$$L=\prod_{i=1}^{N}L_{i},$$

where *L_i_* is defined as in either Section 6.1.1 or 6.2. This procedure can be performed using the R function optim, which along with parameter estimates provides a numerically derived Hessian matrix evaluated at these estimates. For the particular choices of random effects distributions described in Section 6.1.2, it is worth noting that many of the integrations involved in the marginal likelihood can be computed analytically. See the supplemental material in Yiu et al. (2016) for more details.

## 7 Generalized Estimating Equations and Bayesian Approaches to Two-Part Models for Longitudinal Data

GEE approaches have been developed to analyze zero-inflated longitudinal data within the two-part model structure (Moulton et al. 2002, Hall & Zhang 2004, Lu et al. 2004, Yang & Simpson 2010). Population-averaged covariate effects in both parts of the model are directly available from these approaches. One goal in these papers is to avoid the multidimensional integration in maximum likelihood approaches. For this purpose, Bayesian approaches have also been adopted in the literature, but there is no essential difference in the model structures used in these estimation approaches (Zhang et al. 2006, Ghosh & Albert 2009, Neelon et al. 2011, Smith et al. 2015).

## 8 Issues in the Use of Two-Part Models in Longitudinal Settings

### 8.1 Correlated Random Effects and Potential Bias in Estimation

If an assumption of independence between random effects is made, then the likelihood components for the binary and continuous parts of the two-part models for semicontinuous data in Section 3.1 are separable (Tooze et al. 2002). In this case, maximization of the likelihood is computationally simplified. However, if the random effects are correlated, there is an informative cluster size aspect to the data structure because the parameters in the binary part determine the probabilities of nonnegative observations at visit times, and consequently the number of nonnegative observations contributing to the continuous part of the model (Su et al. 2009) for a subject. Essentially, with a positive correlation between *U_i_* and *V_i_*, subjects with larger random effects *U_i_* in the binary part will also have larger random effects *V_i_* in the continuous part, which will translate to them having more observations contributing toward estimation of the continuous part of the model. Moreover, these contributed observations will overrepresent larger values in the continuous part of the data. Because we assume that *E*(*V_i_*) = 0, an incorrect assumption of independence between random intercepts, and the consequent analysis of the continuous part of the data separately from the binary part, will produce positive bias in estimating the intercept term in ***β***. The impact on estimation of other regression coefficients in ***β*** will depend on ***θ***, $\sigma_{u}^{2},\sigma_{v}^{2},$ *ρ*, $\sigma_{e}^{2},$ and the true values for ***β***. The regression parameters ***θ*** remain unbiased under the incorrect assumption of independence between random intercepts (i.e., *ρ* = 0).

This problem was considered by Su et al. (2009), who observed that it parallels conceptually the nonignorable missingness problem characterized in a class of shared parameters models (Wu & Carroll 1988, Wu & Bailey 1989, Henderson et al. 2000, Saha & Jones 2005). The model for the binary part of semicontinuous data corresponds to the logistic random effects model for missing indicators in shared parameters models; the continuous part is similar to the partly unobserved outcome data modeled (typically) by linear mixed models. Underlying random effects in the shared parameters models link the model for missing indicators and the model for outcomes, whereas in our case the shared parameters are exactly those controlling correlated random intercepts (*U_i_*, *V_i_*) in Equation 3. The only difference between these two scenarios is that in two-part mixed models, both ***θ*** and ***β*** are primary targets of inference, whereas in shared parameters models, only ***β*** in the outcome model is of interest.

Su et al. (2009) present results on the asymptotic bias in the estimation of ***β*** in the misspecified two-part mixed models with random intercepts only, assuming that all variance component parameters are known. Let *t_ij_* = 0, 1 denote two measurement times for each subject and *G_i_* = 0, 1 denote a binary covariate, say a treatment indicator. It is further assumed that subjects are equally likely to be assigned to the two treatment groups and that

1. logit{Pr(*Z_ij_* = 1 | *U_i_*)} = *θ*_0_ + *θ*_1_*t_ij_* + *θ*_2_ *G_i_* + *U_i_*,
2. conditional on $Y_{ij}>0,[\text{log}(Y_{ij})\;|\;Y_{ij}>0,V_{i}]∼N(\beta_{0}+\beta_{1}t_{ij}+\beta_{2}G_{i}+V_{i},\sigma_{e}^{2}),$ and
3. (*U_i_*, *V_i_*) follow the bivariate normal distribution as in Equation 3.

The asymptotic bias for estimating ***β*** depends on ***θ*** (or equivalently, the proportion of nonzero values for a typical subject in the treatment groups), the between-subject variability of occurrence variables $\sigma_{u}^{2},$ the between-subject variability of nonzero values $\sigma_{v}^{2},$ and the error variance of nonzero values $\sigma_{e}^{2},$ Given that the other parameters are fixed, in this specific scenario, the bias for ***β*** is independent of the true values of ***β***.

For the simple scenario when *θ*_1_ = −1, *θ*_2_ = log(2), and $\sigma_{e}^{2}$ is fixed at 0.08 (a value derived from analysis of HAQ data), Su et al. (2009) investigate how the asymptotic bias varies as a function of *θ*_0_, $\sigma_{u}^{2},$ $\sigma_{v}^{2},$ and the correlation parameter *ρ*.

**Figure 3:**
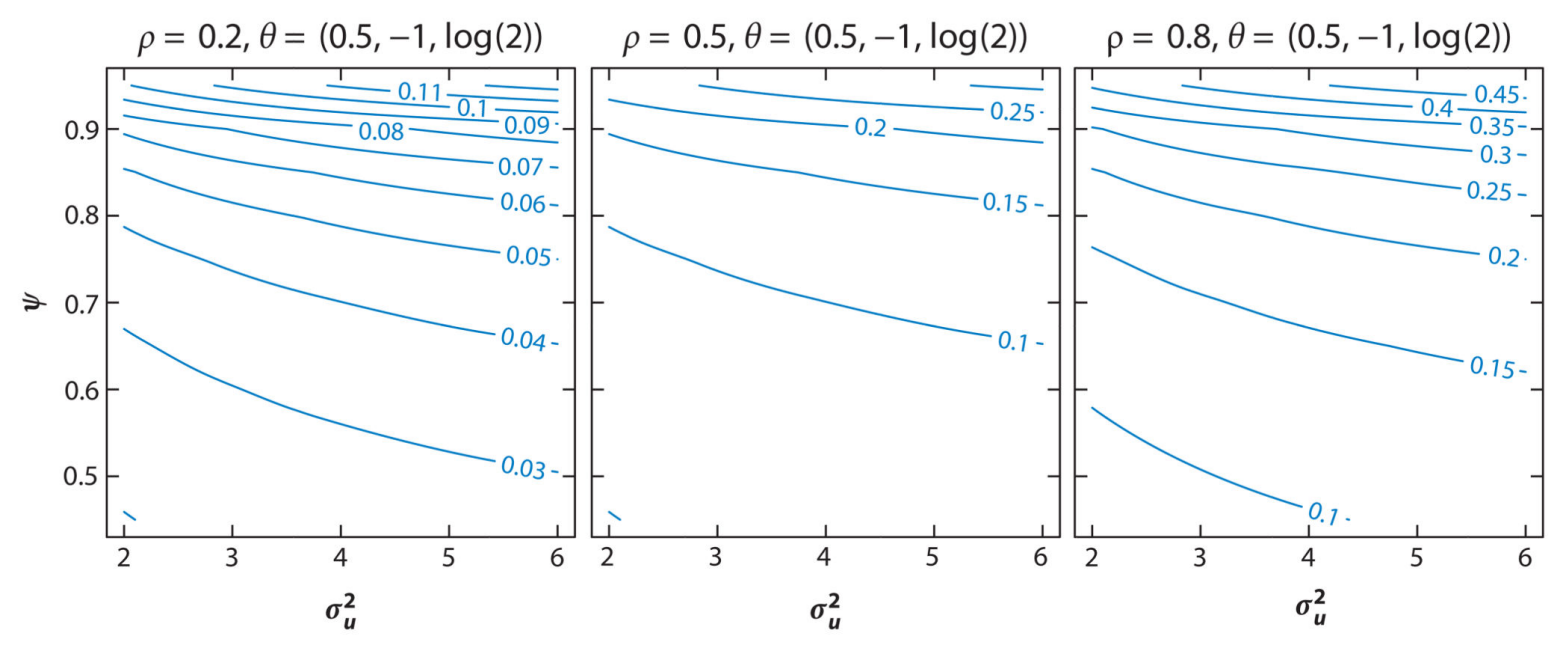
Contour plots of asymptotic bias for the intercept term *β*_0_ in a misspecified two-part mixed model by occurrence random intercept variance $\sigma_{u}^{2}$ and intraclass correlation $\sigma_{v}^{2}/(\sigma_{v}^{2}+\sigma_{e}^{2}),$ stratified by correlation between random effects [*ρ* = (0.2, 0.5, 0.8)] and overall proportion of zeros [i.e., intercept term in the binary part *θ*_0_ = (−0.5, 0.5, 1.5); (*θ*_1_, *θ*_2_) = (−1, log(2)) are fixed]. The error variance is fixed at $\sigma_{e}^{2}=0.08.$ Adapted with permission from Su et al. (2009).

Figure 3, adapted from Su et al. (2009), presents the contour plots of absolute asymptotic bias for estimating the intercept term *β*_0_, plotted according to $\sigma_{u}^{2}$ and the intraclass correlation $\psi =\sigma_{v}^{2}/\left(\sigma_{v}^{2}+\sigma_{e}^{2}\right)$ for different values of *ρ* with *θ*_0_ = 0.5 and *θ*_1_ = log(2). The axes for $\sigma_{u}^{2}$ and *ψ* are centered at 4 and 0.7 respectively, again based on analysis of HAQ data. Figure 3 illustrates that *β*_0_ is overestimated, and the magnitude of the bias is positively related to the correlation parameter *ρ*, the between-subject variability of occurrence variables $\sigma_{u}^{2},$ and the between-subject variability of nonzero values $\sigma_{v}^{2}$ (or equivalently *ψ*). Su et al. (2009) also show that, as *θ*_0_ (the proportion of nonzero values in a control subject) increases, the bias in the estimation of *β*_0_ decreases.

Investigations of the absolute asymptotic bias in estimating the time effect, *β*_1_, and the treatment effect, *β*_2_, show that there is a positive bias for *β*_1_ and a negative bias for *β*_2_, but the bias is much less than that observed for *β*_0_. A more comprehensive discussion of biases is given by Su et al. (2009).

### 8.2 Marginal Inferences in Two-Part Models with Random Effects

The formulation of two-part models for longitudinal data, particularly those with random effects in both parts of the model, often makes the characterization of marginal means, and associated marginal effects of covariates, problematic. We consider this topic in the context of the model for longitudinal semicontinuous data introduced in Section 4.1 and the longitudinal model for zero-inflated count data of Section 5.1, based on work by Su et al. (2015), Tom et al. (2016), and Long et al. (2015).

#### 8.2.1 Marginal means from the binary part of a model for semicontinuous data

If a random effects logistic model is desired for the binary part of a two-part model for semicontinuous data, then a natural choice would be the one specified in Section 4.1, which adopts a bridge density for the distribution of the random effects. Then as outlined there, the subject-specific conditional (on random effect) and population-averaged marginal forms have the same logistic form with regression coefficients proportional to each other. Thus, it is straightforward to summarize inferences in either form as required. Although, to our knowledge, this has not formally been explored, the same formulation of the binary part of a hurdle model for count data would also be possible.

#### 8.2.2 Marginal means from the continuous part of a model for semicontinuous data

Assessment of the impact of a covariate on the marginal mean in the continuous part of a two-part model, $E\left\{g\left(Y_{ij}\right)\;|\;{\text{X}}_{ij}^{*},Y_{ij}>0\right\},$ depends on whether or not that covariate is also involved in the binary part of the two-part model. If the covariate is not included in the binary part or if the random effects *B_i_* and *V_i_* are uncorrelated (i.e., *ρ* = 0), then the interpretation of its effect on $E\left\{g\left(Y_{ij}\right)\;|\;{\text{X}}_{ij}^{*},Y_{ij}>0\right\}$ can be quantified through just the appropriate element of ***β***. However, when *B_i_* and *V_i_* are correlated and, in addition, the covariate of interest is in both regression components of the model, then a simple interpretation is not readily obtainable because of the nonlinearity of $E\left\{g\left(Y_{ij}\right)\;|\;{\text{X}}_{ij}^{*},Y_{ij}>0\right\}$ in this covariate. Specifically, note that the population averaged marginal mean of $g\left(Y_{ij}\right)\;|\;{\text{X}}_{ij}^{*},Y_{ij}>0$ after integrating over (*B_i_*, *V_i_*) is not ${\text{X}}_{ij}^{*}\beta ,$ but

(6) $$E\left\{g\left(Y_{ij}\right)\;|\;{\text{X}}_{ij}^{*},Y_{ij}>0\right\}={\text{X}}_{ij}^{*}\beta +E\left(V_{i}\;|\;{\text{X}}_{ij}^{*},Y_{ij}>0\right),$$

which will be dependent on the impact of covariates ${\text{X}}_{ij}^{*}$ on the marginal and conditional probabilities of occurrence (see the supplemental material of Tom et al. 2016 for more details).

As the integral given by $E\left(V_{i}\;|\;{\text{X}}_{ij}^{*},Y_{ij}>0\right)$ has no closed form solution, an exact analytical expression for Equation 6 is not available. Tom et al. (2016) derived the bounds on Equation 6 as follows: For *ρ* ≥ 0,

$${\text{X}}_{ij}^{*}\beta \leq E\left(g\left(Y_{ij}\right)\;|\;{\text{X}}_{ij}^{*},Y_{ij}>0\right)\leq {\text{X}}_{ij}^{*}\text{β}+\frac{\sigma_{v}\rho }{\sqrt{2\pi }}\left(1+e^{-{\text{X}}_{ij}\text{θ}}\right),$$

and for *ρ* ≤ 0,

$${\text{X}}_{ij}^{*}\beta \geq E\left(g\left(Y_{ij}\right)\;|\;{\text{X}}_{ij}^{*},Y_{ij}>0\right)\geq {\text{X}}_{ij}^{*}\beta +\frac{\sigma_{v}\rho }{\sqrt{2\pi }}\left(1+e^{-{\text{X}}_{ij}\theta }\right).$$

Although an exact analytical expression is not available, numerically solving Equation 6 at the maximum likelihood estimates is straightforward, as only a single integral is involved. This integral can be evaluated using adaptive Gaussian quadrature techniques, where the parameters ***θ***, ***β***, $\sigma_{b}^{2},$ $\sigma_{v}^{2},$ $\sigma_{e}^{2},$ and *ρ* are replaced by their maximum likelihood estimates.

Subsequently, the impact of a covariate could be assessed through plotting the relationship between this covariate and $E\left(Y_{ij}\;|\;{\text{X}}_{ij}^{*},Y_{ij}>0\right),$ with other covariates held fixed, or alternatively by describing the local changes (i.e., through the derivative or the difference) in $E\left(Y_{ij}\;|\;{\text{X}}_{ij}^{*},Y_{ij}>0\right)$ with respect to the covariate (Liu et al. 2010). However, the clinical relevance of $E\left(Y_{ij}\;|\;{\text{X}}_{ij}^{*},Y_{ij}>0\right)$ has been questioned, as discussed by Albert et al. (2005), in light of work by Lu et al. (2004) and Williamson et al. (2003). For example, in the context of the HAQ data for PsA patients, the patients whose data contributed to the estimation of $E\left(Y_{ij}\;|\;{\text{X}}_{ij}^{*},Y_{ij}>0\right)$ are different over time. Therefore, in this case, it is questionable whether the targeted population is meaningful when the marginal inference of covariate effects is based on $E\left(Y_{ij}\;|\;{\text{X}}_{ij}^{*},Y_{ij}>0\right).$ The overall marginal mean of *Y_ij_* as the target of inference is more easily justified clinically, as discussed in Section 9.4, with respect to the association between HLA alleles and overall expected disability level.

#### 8.2.3 Overall marginal mean

When *g*(·) is the identity function, the overall marginal mean of the response $E(Y_{ij})\equiv E(Y_{ij}\;|\;{\text{X}}_{ij},{\text{X}}_{ij}^{*})$ is given by

$$E(Y_{ij}\;|\;Y_{ij}=0)\text{Pr}(Y_{ij}=0)+E(Y_{ij}\;|\;Y_{ij}>0)\text{Pr}(Y_{ij}>0)=E(Y_{ij}\;|\;Y_{ij}>0)\text{Pr}(Y_{ij}>0),$$

where we have suppressed the dependence on the covariate vectors, ${\text{X}}_{ij},{\text{X}}_{ij}^{*},$ for convenience. Although a closed form for the overall marginal mean is not available, it can be evaluated numerically.

Using previous results on bounds for the conditional marginal mean, bounds on the overall marginal mean can be obtained as

$$\text{Pr}(Y_{ij}>0){\text{X}}_{ij}^{*}\beta \leq E(Y_{ij})\leq \text{Pr}(Y_{ij}>0){\text{X}}_{ij}^{*}\beta +\frac{\sigma_{v}\rho }{\sqrt{2\pi }}$$

when *ρ* ≥ 0, and

$$\text{Pr}(Y_{ij}>0){\text{X}}_{ij}^{*}\beta \geq E(Y_{ij})\geq \text{Pr}(Y_{ij}>0){\text{X}}_{ij}^{*}\beta +\frac{\sigma_{v}\rho }{\sqrt{2\pi }}$$

when *ρ* ≤ 0. Note that Pr(*Y_ij_* > 0) = (1 + *e*^−**X**_*ij*_**θ**^)^−1^.

Similar bounds can be derived for other common monotonic transformation functions for *g*(·). For example, the bounds on the overall marginal mean when *g*(·) is logarithmic are provided by Tom et al. (2016).

#### 8.2.4 Marginalized model

We have highlighted some of the challenges when making marginal inferences from two-part models in the context of longitudinal semicontinuous data. Many of these issues are similar for ZIP models for longitudinal count data. In particular, inferences on the overall mean, rather than the means in the two-part structure, are desirable in certain clinical settings, as illustrated in Section 10.1 using the SafeTalk data. In this section, we focus on a different approach that involves formulating directly a regression model for the overall mean of longitudinal count data arising from a ZIP structure.

For the ZIP model of Section 5.1, the overall conditional (subject-specific) mean $E(Y_{ij}\;|\;{\text{X}}_{ij},{\text{X}}_{ij}^{*},U_{i},V_{i})=\{1-\mathrm{Pr}(Z_{ij}=\text{0}\;|\;{\text{X}}_{ij},U_{i})\}\mu_{ij}^{C}$ will depend on ***θ***, ***β*** and *U_i_*, *V_i_* through a complicated function, which makes it difficult to evaluate covariate effects on the overall conditional mean. Specifically, if one is interested in a particular covariate effect, then this can only be examined by fixing other covariates in order to define the necessary transformations and allow variance estimation. To avoid this difficulty, Long et al. (2015) suggested the alternative model that, rather than modeling the conditional Poisson mean $\mu_{ij}^{C},$ models the overall conditional mean $\nu_{ij}^{C}=E(Y_{ij}\;|\;{\text{X}}_{ij},{\text{X}}_{ij}^{*},W_{i})$ through

(7) $$\text{log}\left(\nu_{ij}^{C}\right)={\text{X}}_{ij}^{*}\alpha +\text{log}(O_{ij})+W_{i},$$

where the random intercepts, (*U_i_*, *W_i_*), are mean zero normal variables with variances $\sigma_{u}^{2}$ and $\sigma_{w}^{2}$ and correlation *ρ*, and log(*O_ij_*), an offset variable, has again been introduced to allow for situations where the incidence density *ν_i_*/*O_ij_* is of interest. The use of the term marginalized, rather than marginal, for this model is adopted because it is a subject-specific marginal mean, conditional on a subject’s random effects, that is being modeled and not a population-averaged mean, which marginalizes over random effects. However, as shown by Long et al. (2015), for all fixed covariates that do not have corresponding random effects, the subject-specific parameters in Equation 7 are equivalent to population-averaged parameters.

Because $\nu_{ij}^{C}$ is modeled directly in this marginalized ZIP model with random effects, the *k*th parameter of ***α***, *α_k_*, is interpreted as the subject-specific log-incidence density ratio (IDR) for the *k*th covariate; that is, for a one-unit increase in corresponding covariate $x_{ijk}^{*},$ exp(*α_k_*) is the amount by which the mean $\nu_{ij}^{C}$ for a particular subject is multiplied, which is the same interpretation as in a Poisson random effects model. The direct modeling of $\nu_{ij}^{C}$ rather than the Poisson mean $\mu_{ij}^{C}$ provides inference for the subject-specific overall mean.

The log-likelihood for this marginalized ZIP model with random effects can be written, in a similar manner to that given in Section 5.2, as

(8) $$L=\prod_{i=1}^{N}\int_{u_{i}}\int_{w_{i}}\prod_{j=1}^{n_{i}}\text{Pr}(y_{ij}\;|\;\theta ,\alpha ,u_{i},w_{i})f(u_{i},w_{i}\;|\;\sigma_{u}^{2},\sigma_{w}^{2},\rho )\text{d}w_{i}\text{d}u_{i},$$

where

$$\begin{array}{l} \text{Pr}(y_{ij}\;|\;\theta ,\beta ,u_{i},w_{i})={\left[\text{Pr}(Z_{ij}=0\;|\;{\text{X}}_{ij},u_{i})+(1-\text{Pr}(Z_{ij}=0\;|\;{\text{X}}_{ij},u_{i}))e^{-\mu_{ij}^{C}}\right]}^{1-z_{ij}}\\ \;\times {\left[\frac{(1-\text{Pr}(Z_{ij}=0\;|\;{\text{X}}_{ij},u_{i}))e^{-\mu_{ij}^{C}}{\left(\mu_{ij}^{C}\right)}^{y_{ij}}}{y_{ij}!}\right]}^{z_{ij}}. \end{array}$$

However, unlike in Section 5.1, for this marginalized model, $\mu_{ij}^{C}=\text{exp}(\delta_{ij}^{C}),$ where $\delta_{ij}^{C}$ is not necessarily a linear function of covariates. In particular, using the defining model equations and the knowledge $\nu_{ij}^{C}=\{1-\text{Pr}(Z_{ij}=0\;|\;{\text{X}}_{ij},U_{i})\}\mu_{ij}^{C},$ solving for $\delta_{ij}^{C}$ gives

(9) $$\delta_{ij}^{C}=\text{log}(O_{ij})+\text{log}\{1+\text{exp}(-{\text{X}}_{ij}\theta -U_{i})\}+{\text{X}}_{ij}^{*}\alpha +W_{i}.$$

Through substitution of Equation 9 into Equation 8, this subject-specific marginalized ZIP model with random effects may be fit using SAS NLMIXED, which employs an adaptive Gauss-Hermite quadrature to approximate the integral of the likelihood over the random effects. Additionally, SAS NLMIXED can provide robust (empirical) standard error estimates of the parameters, through the likelihood-based sandwich estimator, to address model misspecification (White 1982). Following Long et al. (2015), these robust estimates are emphasized in the analysis of the SafeTalk data in Section 10.

Preisser et al. (2016) have extended the marginalized ZIP model to account for overdispersion in addition to excess zeros through a marginalized ZINB, though only for cross-sectional data. In addition, marginalized two-part models for semicontinuous data have been developed by Smith et al. (2014, 2015) for both cross-sectional and longitudinal settings. They noted that under this type of model specification, the regression parameters for the binary part of the model contribute to the likelihood for the continuous part so that some degree of correlation is inherently included between the two components of the model. Therefore, the parameter *ρ* is, for this model, allowing for additional correlation due to any unobserved processes that influence both the probability of a nonzero value and the overall mean for a subject.

### 8.3 The Concept of Two Populations

Early work on two-part models for survival data, explicitly or implicitly, often involved the concept of a population of cured patients, or at least a separate population of long-term survivors. The natural question when using such models was, therefore, is there evidence for this separate population?

In many applications of two-part models for longitudinal data, this type of question need not arise. For example, in the two-part models for longitudinal semicontinuous data and zero-inflated count data, the binary variable that indicates whether an observation is a zero or from an alternative distribution is defined for each observation of a single subject. Generally, in such a situation, and even in cross-sectional settings, this two-part model structure can be seen as a convenient empirical approach to describing data that, for whatever reason, has a preponderance of zeros that cannot adequately be reflected in another manner.

However, in models such as those discussed in Section 6, there is the concept of two populations of subjects, one for which all longitudinal observations are always zero and one for which the observations are all nonzero or a mixture of zeros and nonzeros. A special case of these models corresponds to a version of zero-inflated count data models for longitudinal data that differ from those in Section 5 by having this one-time subject-level specification of the binary indicator for zero rather than a longitudinal set of binary indicators. In this type of model, it is assumed that all observations for some patients must be zero, which makes the concept of a separate population of patients almost unavoidable.

In the context of survival data, Farewell (1986) discusses the risks of adopting such a model and highlights the inferential challenges of providing evidence for or against a separate population. In particular, there can be considerable indeterminacy between the estimation of the probability of being in a separate population and the location parameters of the time-to-event distribution adopted for the other part of the model. This indeterminacy can often be seen through the shape of a profile likelihood function.

With longitudinal data, in some contexts, there might be extra information in the data that can identify such a separate population. For example, in the case of dietary data, if subjects provide the information that they are never-consumers of a type of food, then we can incorporate such information on this separate population into the model. In other scenarios of longitudinal data, it is a reasonable conjecture that there might be more information on the possible existence of a separate population when subjects can be observed to have a long sequence of zero observations. Nevertheless, such information might not be available, and inferences concerning a separate population might strongly depend on the particular form of two-part model adopted. In Section 11, we illustrate how the examination of different two-part models, the shape of likelihood functions, and goodness-of-fit investigations might inform such inferences.

## 9 Analyses of Semicontinuous Health Assessment Questionnaire Data

### 9.1 Potential Bias with a Misspecified Model for Random Effects

The HAQ data described in Section 2.1 can be modeled using a two-part mixed model, and we present results extracted from Su et al. (2009). The random-intercept logistic model (Equation 1) is used to model a binary indicator of a nonzero HAQ score, and the random-intercept linear mixed model (Equation 2) is used for nonzero HAQ scores. No transformation is applied to the nonzero HAQ scores.

The same set of covariates is included in both model parts, but the coefficients are allowed to differ. These covariates include age at onset of PsA (standardized), sex, PsA disease duration in years, total number of actively inflamed joints, total number of clinically damaged joints, Psoriasis Area and Severity Index (PASI) score (standardized), morning stiffness (coded as either present or absent), standardized erythrocyte sedimentation rate, and highest medication level ever used prior to a visit, grouped based on a medication pyramid (Gladman et al. 1995, Munro et al. 1998). Because there is particular interest in differential effects of both the number of actively inflamed joints and the number of clinically deformed joints on physical functioning over the duration of PsA, interaction terms for the duration of PsA with both of these variables are also included in the model.

We refer to the two-part mixed model with correlated random intercepts as the full model. Following (Su et al. 2009), if these data are modeled with an assumption of independent random intercepts, we term it the misspecified model. Selected results from Su et al. (2009) are given in Table 1, which only includes results for the primary covariates of interest.

As shown in Table 1, the estimated coefficients in the binary part are approximately the same in both the full model and the misspecified model, and suggest the same predictors of functional difficulty. Particularly, there is no differential effect of actively inflamed joints on functioning difficulty over the duration of PsA, but there is some evidence that the effect of the number of deformed joints increases with disease duration. The parameter estimates for the distribution of the random intercepts in the binary part are also similar.

As expected from the discussion in Section 8.1 and simulation results in Su et al. (2009), Table 1 shows that, for the continuous part, the misspecified model overestimates the intercept term and underestimates the time-invariant sex effect. For other time-varying covariates, the estimates are approximately the same, except that the coefficients for the PASI score and the interaction between clinically deformed joints and PsA duration are larger in the full model, with correspondingly smaller *p*-values. The random intercept variance in the misspecified model is underestimated, and error variance estimates are similar, consistent with the simulation results in Su et al. (2009). Thus, the qualitative conclusions do not change across models. In particular, the positive effects of active joints and deformed joints differ over the duration of PsA: The effect of the former decreases, while the effect of the latter increases over time.

The deviance and Akaike information criterion values in Table 1 indicate that the full model provides a better fit to the data. A likelihood ratio test of the hypothesis of zero correlation generates a significance level less than 0.0001.

The estimated correlation between random intercepts of the two parts of the full model is positive and close to one (*ρ̂* = 0.94) This suggests that there might be a single unmeasured latent process that influences the two processes, corresponding to perfectly correlated random intercepts. Therefore, a latent process two-part model, such that the correlated random intercepts follow *V_i_* = *αU_i_* and $\sigma_{v}^{2}=\sigma^{2}\sigma_{u}^{2},$ could be fit. The estimates from such a model are very similar to those from the full model.

### 9.2 Marginal Covariate Effects

A focus on marginal covariate effects is perhaps particularly natural with time-invariant variables. As mentioned in Section 2.1, for patients with PsA, there is a particular interest in genetics and the role of alleles that code for HLA. We illustrate the use of the alternative two-part model of Section 4.1 to examine the relationship between the HLA system and physical functioning as measured by the HAQ.

### 9.3 Marginal Covariate Effects for the Binary Part

Some results for the estimated effects of genetic markers (taken from Su et al. 2015) are given in Table 2. The covariates in both parts of the model, **X***_ij_* and ${\text{X}}_{ij}^{*}$ coincide. In this model, age at onset of PsA, sex, and PsA disease duration were also controlled for in both parts. The conditional estimates associated with the binary part of the underlying two-part mixed model, from which the two-part marginal model is derived, are also shown. These conditional effect estimates are obtained by inflating the corresponding marginal covariate effects in the binary part by the reciprocal of *ϕ* = 0.4861 [95% confidence interval (CI): 0.4256–0.5465]. The corresponding standard errors were calculated using the delta method.

From Table 2, we observe that the presence of *HLA-B27* (HLA allele B27) significantly increases both the odds of the presence of functional disability (*p* = 0.0324) and the actual level of physical functioning given that one has functional disability (*p* = 0.0294). The (marginal) odds ratio associated with *HLA-B27* is 1.605 (95% CI: 1.041–2.476) and the subject-specific difference in the mean (nonzero) HAQ scores between PsA patients with *HLA-B27* present compared with PsA patients with *HLA-B27* absent, but all else the same, is 0.1652 (95% CI: 0.0166–0.3138). Furthermore, there is statistically significant evidence (*p* = 0.0358) for an interaction effect between *HLA-DQw3* and *HLA-DR7* on the probability of having functional disability, with an apparent detrimental effect of having *HLA-DQw3* present (compared with absent) whilst in the presence of *HLA-DR7*. There are no statistically significant effects of *HLA-DQw3*, *HLA-DR7*, or their interaction on the level of physical functioning once functional disability occurs.

### 9.4 Marginal Covariate Effects for the Continuous Part and for the Overall Mean

Whereas the third column of Table 2 presents the conditional covariate effects, given random effects, in the continuous part of this two-part model, as noted in Section 8.2.2, the corresponding marginal covariate effects are generally not equal to these conditional effects. However, as also noted in Section 8.2.2, it is perhaps more natural to examine the association between the HLA alleles and the overall expected disability level of the patients over the study period, instead of the association when some disability is present. This is because disability as measured by the HAQ for patients can vary over time and, for example, at one visit a patient can have mild disability, but at the next visit his or her situation may be improved, resulting in a zero value of HAQ. Thus, it might be considered clinically more informative to present the marginal covariate effects on the overall expected disability level together with the marginal covariate effects on the probability of having any level of disability.

This can be done by sampling from the asymptotic distribution of the parameters based on the estimates in Table 2 and calculating the contrasts of overall expected HAQ with and without specific HLA alleles, controlling for other covariates. In particular, we might fix the age at PsA diagnosis at 35 years and disease duration at 15 years, which correspond to zero values in standardized versions of the two variables. These contrasts represent the effects of HLA alleles on the overall expected disability level (controlling for other covariates) in the PsA cohort.

Because the overall mean of the HAQ score is not directly parameterized in the fitted model, the corresponding covariate effects are not the same for all values of the other variables. Nevertheless, the *HLA-B27* effects (not shown) are approximately the same across different combinations of other covariates, and the 95% CIs do not include zero.

Recall that a significant interaction between the effects of *HLA-DQw3* and *HLA-DR7* was seen in the binary part of the two-part mixed model (*p* = 0.035), whereas the same interaction was nonsignificant in the continuous part (*p* = 0.85). The estimated marginal (log-odds ratio) effect of this interaction in the binary part was 0.8089 with 95% CI [0.0565, 1.5613].

**Figure 4:**
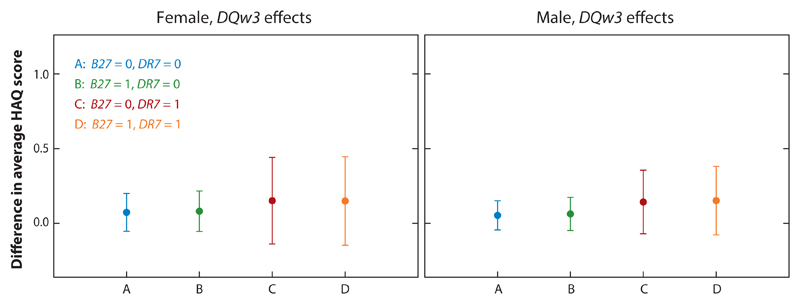
Contrasts (with 95% confidence intervals) of the human leukocyte antigen (HLA) allele *HLA-DQw3* effects on the overall mean of the Health Assessment Questionnaire (HAQ) scores for different combinations of the HLA alleles *HLA-B27* and *HLA-DR7* (controlling for being 35 years old at psoriatic arthritis diagnosis and having a disease duration of 15 years). Adapted with permission from Tom et al. (2016).

Figure 4 reflects this possible interaction between the marginal effects of *HLA-DQw3* and *HLA-DR7* on the overall marginal mean of HAQ stratified by gender and the absence/presence of the *HLA-B27* allele. Again, age at PsA diagnosis is fixed at 35 years and disease duration at 15 years.

Consider the left panel of Figure 4. For females with the presence of *HLA-B27*, we estimate that the difference in the *HLA-DQw3* effects on the overall marginal mean of HAQ between those with the presence of *HLA-DR7* allele and those with it absent (i.e., contrast *D* − *B* in the figure) is 0.0564 with 95% CI [−0.2062, 0.3232]. For females with *HLA-B27* absent, the estimate of this difference in the *HLA-DQw3* effects on the overall marginal mean of HAQ between those with and without the *HLA-DR7* allele (i.e., contrast *C* − *A*) is 0.0648 with 95% CI [−0.1971, 0.3158]. These estimates of the *HLA-DQw3* and *HLA-DR7* interaction for females, with and without *HLA-B27* present, are similar and both statistically nonsignificant. Exactly the same results could be presented by a comparable plot of *HLA-DR7* effects. Conclusions based on these results are thus similar to those found for the continuous part of the two-part marginal model.

## 10 Evaluating the Motivational Interview Intervention in the Safetalk Study

As outlined in Section 2.3, for the clinical trial examining the efficacy of the SafeTalk intervention, participants were randomized to receive either SafeTalk intervention counseling or a control nutritional counseling. The primary count outcome of interest was UAVI. Participants at three study sites completed questionnaires about both nutritional and sexual behavior at baseline as well as at three follow-up visits spaced at four-month intervals. After data cleaning, the sample sizes at each time point were 476, 399, 363 and 301. The overall percentage of zero UAVI counts across both treatment groups and all visits was 83.1%.

### 10.1 Marginalized Zero-Inflated Poisson Models with Random Effects

In order to evaluate the efficacy of the SafeTalk intervention over time, the marginalized ZIP with random effects of Equation 7 was fit by Long et al. (2015) to the UAVI counts at all four time points. The model of interest is

$$\begin{array}{l} \text{logit}\left(\text{Pr}\left(Z_{ij}=1\right)\right)=\theta_{0}+\theta_{1}x_{i1}+\theta_{2}x_{i2}+\theta_{3}I\left(j=2\right)+\theta_{4}I\left(j=2\right)g_{i}\\ \;+\theta_{5}I\left(j=3\right)+\theta_{6}I\left(j=3\right)g_{i}+\theta_{7}I\left(j=4\right)+\theta_{8}I\left(j=4\right)g_{i}+U_{i},\\ \;\text{log}\left(v_{ij}^{C}\right)=\alpha_{0}+\alpha_{1}x_{i1}^{*}+\alpha_{2}x_{i2}^{*}+\alpha_{3}I\left(j=2\right)+\alpha_{4}I\left(j=2\right)g_{i}\\ \;+\alpha_{5}I\left(j=3\right)+\alpha_{6}I\left(j=3\right)g_{i}+\alpha_{7}I\left(j=4\right)+\alpha_{8}I\left(j=4\right)g_{i}+W_{i}, \end{array}$$

where *j* is the visit number, *g_i_* is an indicator of randomization to SafeTalk intervention group, *x*_*i*1_ and *x*_*i*2_ and the identically defined $x_{i1}^{*}$ and $x_{i2}^{*}$ are indicator variables specifying study site, and *U_i_* and *W_i_* follow the bivariate normal distribution as in Section 8.2.4.

The results of an analysis of the SafeTalk trial data are presented in Table 3 (adapted from Long et al. 2015). The contrast testing treatment effect over time *H*_0_ : (*α*_4_, *α*_6_, *α*_8_)′ = (0, 0, 0)′ is highly significant (Wald-type robust *p* = 0.0003), indicating that the SafeTalk intervention affects UAVI count. At the second follow-up visit, for which the IDR (and 95% Wald-type robust CI) is 0.542 (0.260, 1.128), a specific participant randomized to SafeTalk has 46% fewer unprotected sexual acts with any partner than *he or she* would have if randomized to the nutritional intervention. Because the only random effect for the above model is a random intercept, the parameters associated with treatment effect from this analysis additionally have population-averaged interpretations. Thus, at the second follow-up visit, *those participants* randomized to SafeTalk had on average 46% fewer unprotected sexual acts with any partner than *the participants* randomized to the nutritional intervention. The SafeTalk intervention appears to have the largest effect on UAVI count at the first follow-up survey, where the estimated IDR (and 95% Wald-type robust CI) of treatment effect is 0.280 (0.145, 0.542). By the third follow-up survey, we observe less reduction in UAVI count due to SafeTalk, with an IDR of 0.769 (0.307, 1.928). Some reduction in predicted UAVI count can also be seen in the nutritional control arm at the final visit, numerically represented through *α*_7_. Additionally, note that the correlation between the random intercepts, estimated to be −0.79, is highly significant, indicating those participants with higher expected UAVI counts have lower odds of excess zero latent class membership. In fact, if independence of the random intercepts is assumed, individual parameter estimates from the marginalized ZIP model differ by as much as 40% (results not shown), demonstrating the same type of bias discussed in Section 8.1.

### 10.2 Comparison with Traditional Zero-Inflated Poisson Models with Random Effects

To highlight the differences between the proposed marginalized ZIP model with random effects and the ZIP model with random effects from Section 5.1, the latter model was also fit by Long et al. (2015) to the SafeTalk data, with the model given by

$$\begin{array}{l} \text{log}\text{it}\left(\text{Pr}\left(Z_{ij}=1\right)\right)=\theta_{0}+\theta_{1}x_{i1}+\theta_{2}x_{i2}+\theta_{3}I\left(j=2\right)+\theta_{4}I\left(j=2\right)g_{i}\\ \;+\theta_{5}I\left(j=3\right)+\theta_{6}I\left(j=3\right)g_{i}+\theta_{7}I\left(j=4\right)+\theta_{8}I\left(j=4\right)g_{i}+U_{i},\\ \;\text{log}\left(\mu_{ij}^{c}\right)=\beta_{0}+\beta_{1}x_{i1}^{*}+\beta_{2}x_{i2}^{*}+\beta_{3}I\left(j=2\right)+\beta_{4}I\left(j=2\right)g_{i}\\ \;+\beta_{5}I\left(j=3\right)+\beta_{6}I\left(j=3\right)g_{i}+\beta_{7}I\left(j=4\right)+\beta_{8}I\left(j=4\right)g_{i}+V_{i}, \end{array}$$

where *U_i_* and *V_i_* follows the bivariate normal distribution as in Equation 3. For this model, the contrast of treatment effect is highly significant (*p* < 0.0001) with *β*_4_ = −0.96, *β*_6_ = −0.89, and *β*_8_ = −0.42. In contrast to the marginalized ZIP model with random effects, these traditional ZIP parameter estimates are the log-IDR for treatment among the non-excess zero latent class. Thus, among the non-excess zero latent class, those participants randomized to SafeTalk had 62%, 59% and 35% fewer UAVI acts than those participants randomized to control at the first, second, and third follow-up visits, respectively.

## 11 Mover-Stayer Models for Damage in Psoriatic Arthritis

As indicated in Section 2.2, joint damage is often used as a measure of disease progression in PsA. Several authors (Aguirre-Hernández & Farewell 2004, Solis-Trapala & Farewell 2005, and O’Keeffe et al. 2012) have considered the existence of a subpopulation of patients who do not have the propensity to experience clinical joint damage. The mover-stayer model of Section 6.1.2 also provides a framework to examine the possibility of such a subpopulation. The model would assume that there are two populations of patients, stayers who have no risk of damage and movers who are at risk. This approach is illustrated in this section, with the development following that in Yiu et al. (2016).

The data collected are counts of damaged joints, made at each clinic visit, where damage is considered to represent a permanent change, and therefore the count of damaged joints cannot decrease over time. The count variable of interest, denoted *Y_ij_* in Section 6.1.2, is the change in damaged joint counts between clinic visits at times *t*_*ij*−1_ and *t_ij_* (*j* = 1,…, *n_i_* and *t*_*i*0_ = 0); log(*O_ij_*) = log(*t_ij_* − *t*_*ij*−1_) is the offset and *X_ij_* are study entry or lagged-one (i.e., previous visit) covariate information. To produce a homogeneous set of patients, the data are restricted to the 28 hand joints, 14 in each hand, and to 757 patients who entered the University of Toronto PsA Clinic with no damaged hand joints and had more than one clinic visit. The mean and median numbers of clinic visits per patient were 11.27 and 7, and the number of clinic visits ranged from 2 to 57. The mean follow-up time was 9.46 years, with an interquartile range of 11.15 years. The mean and median inter-visit times were 0.84 and 0.54 years, with a standard deviation (SD) of 1.19 years. There were 232 patients who entered the clinic with damaged hand joints and had more than one clinic visit. Although on average 7 years older at clinic entry [mean age (SD): 49.07 (12.62) years versus 42.19 (12.48) years], these 232 patients were not that different in gender distribution, age at arthritis onset, follow-up and inter-visit times and number of clinic visits than the 757 patients considered with undamaged joints. However, these patients, not surprisingly, had on average higher numbers of disease-active hand joints [mean active joint count (SD): 6 (6) joints versus 2.1 (3.7)].

While in the clinic, a large percentage, 72% (524 patients), of the 757 patients remained damage free in the hand joints. Of the patients (233 patients) who developed damaged joints, the mean rate of gaining damage was 0.53 joints per year. Although the development of damaged hand joints is not formally a recurrent events process (because there is a finite number of hand joints), the models for the movers are based on Poisson processes, as an approximation, because there are few occasions when a large number of damaged hand joints have been observed.

### 11.1 Poisson Mover-Stayer Models

Table 4 presents the results of fitting Poisson M-S models with the three random effects distributions given in Section 6.1.2. The covariates included in the Poisson component of the model included the numbers of damaged and active (painful or swollen) joints at the previous visit, arthritis duration, and age at onset of arthritis, all known from other studies to be related to the risk of developing damaged joints.

For the Poisson M-S models, the gamma and inverse Gaussian distributional parts of the mover-stayer random effects distributions were parameterized to have unit means in order to avoid identifiability problems with the baseline intensity. An alternative but mathematically equivalent approach to avoid nonidentifiability was taken for the CP random effects distribution, which has an expectation of $\frac{\rho_{i}}{v};\rho_{i}$ and *ν* were allowed to vary freely on ℝ^+^ with λ_0_ constrained to unity.

All models were fitted with *π_i_* = *π* so that the existence of a stayer population could be more simply investigated, specifically through testing the null hypothesis *H*_0_ : *π* = 0 for the Poisson M-S gamma, Poisson M-S inverse Gaussian, and zero-inflated models. Under the null hypothesis, the asymptotic distribution of the likelihood ratio test for these models (against their non-M-S counterpart) is a 50:50 mixture of a point mass at zero and a $\chi_{1}^{2}$ (Self & Liang 1987). A test of *H*_0_ : *π* = 0 for the Poisson M-S CP model is equivalent to testing *H*_0_ : exp(−*ρ*) = 0 (or *H*_0_ : *ρ* = ∞). However, under this null hypothesis, the parameter *ν* becomes irrelevant, which therefore results in the asymptotic distribution of the likelihood ratio statistic being intractable. For this model, we focus on the 95% Wald interval of *π*ˆ in order to examine the possible existence of a stayer population. Note that models could be easily extended so that *π_i_* depends on covariates, as in the two-part models of Section 5.

For all three models, the regression coefficient estimates are quite similar; most estimates lie in the corresponding 95% Wald interval of the other models. The current number of active joints, arthritis duration, and age at onset of arthritis demonstrate significant positive associations, whereas the current number of damaged joints demonstrates a significant negative association with damage progression. After accounting for correlation through the multiplicative patient-level random effect, one can postulate that the negative association indicates that fewer joints have the propensity to become damaged. This was investigated by Yiu et al. (2016), who obtained similar results with the use of a truncated Poisson distribution. The previous number of damaged joints was primarily introduced by Yiu et al. (2016) to provide information about the history of a patient and therefore introduces correlation between the patient observations. As the patient-level random effect is also designed to partly reflect this type of correlation (in addition to capturing time-invariant unobserved heterogeneity), the effect of this dynamic covariate will likely be confounded with the random effects (see Aalen et al. 2008).

**Figure 5:**
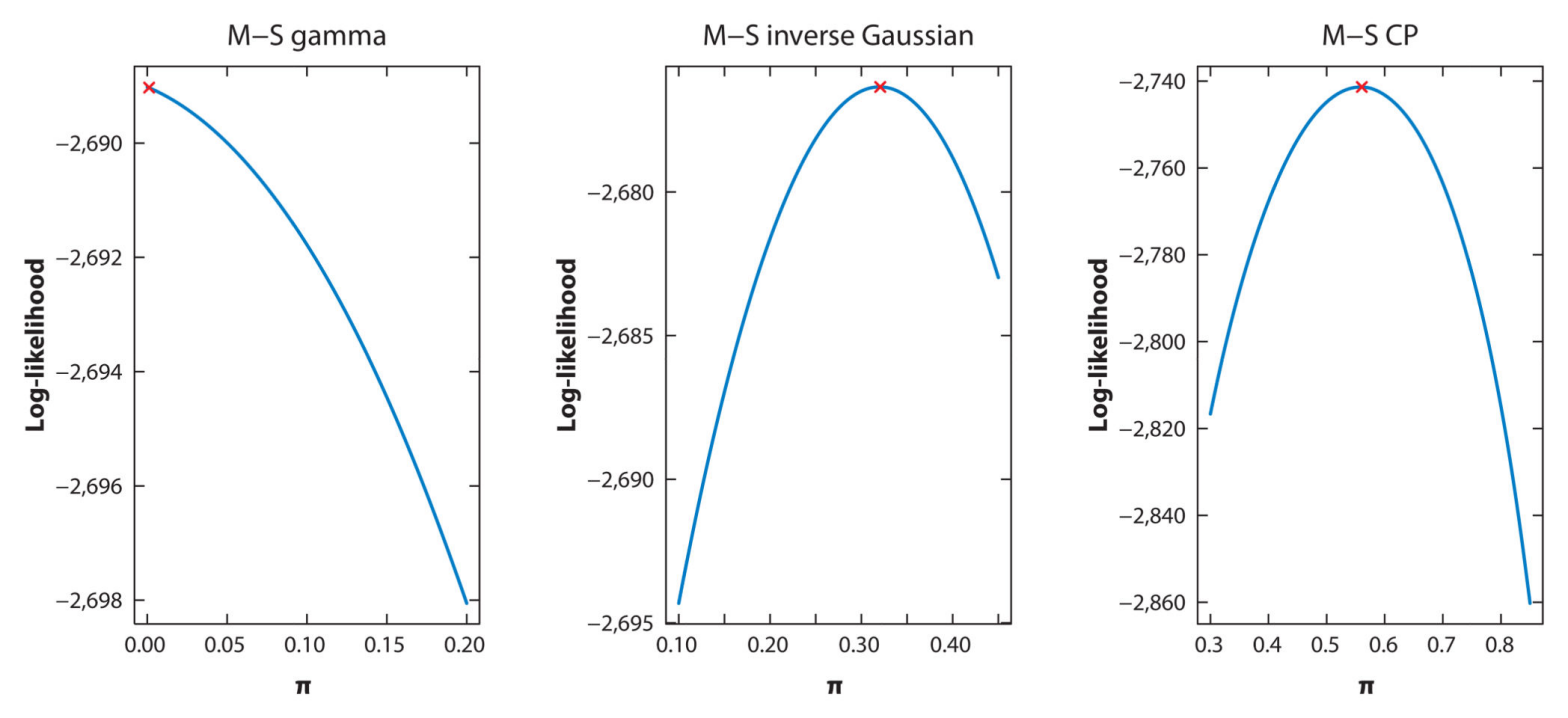
Plots of the profile log-likelihoods for *π*. The cross indicates the point at which the numerical optimization procedure converged. Abbreviations: CP, compound Poisson; M-S, mover-stayer. Adapted with permission from Yiu et al. (2016).

Figure 5 shows plots of the profile log-likelihoods for *π*. From the first panel of the figure, the profile log-likelihood for the Poisson M-S gamma model is seen to be a monotonically decreasing function, which implies that the maximum is attained at the boundary, in particular at *π* = 0, and not at the value produced from the numerical optimization procedure (reported in Table 4). The Poisson M-S gamma model therefore gives no evidence of a stayer population and may even suggest that such a population is unlikely. As the optimization procedure did not converge at the maximum, a more relevant CI (as opposed to a Wald interval) can be computed from the profile likelihood based on values of *π* in which the null hypothesis *H*_0_ : *π* = 0 cannot be rejected. Such an interval was calculated as (0, 0.063). The optimization routine for the other two mover-stayer models (Poisson M-S inverse Gaussian and CP models) do converge at the maximums of their respective profile log-likelihoods. Furthermore, as the stayer proportions and their respective CIs are estimated far from zero, these models are more consistent with a stayer population. A likelihood ratio test of *H*_0_ : *π* = 0 resulted in *p* < 0.001 for the Poisson M-S inverse Gaussian and zero-inflated models, indicating convincing evidence for a stayer population. From Table 4, it can, however, be seen that the stayer proportion estimates vary widely across these models. In particular, the Poisson M-S inverse Gaussian and CP models estimate the stayer proportion as 0.32 (0.2, 0.47) and 0.56 (0.51, 0.61), respectively.

#### 11.1.1 Comments on estimation of *π*

The widely varying estimates of *π* may suggest that the PsA data contained many patients who were slow-transitioning movers and that the fitted models distinguished very differently between slow-transitioning movers and stayers. Of the fitted models, only the Poisson M-S gamma model is consistent with the absence of a stayer population, because *π̂* = 0 Under the current parameterization for *U_i_* Λ_*ij*_, if *θ* > 1, the gamma distribution is such that *g*(*u_i_*) → ∞ as *u_i_* → 0, and therefore this distribution is able to place a large proportion of mass arbitrarily close to zero. Implicitly, the gamma distribution is able to represent a large proportion of patients with slow transition intensities, namely the slow-transitioning movers. In the motivating example, the fitted Poisson M-S gamma model was such that *θ̂* = 6.19(5.11, 7.5) with a slow average estimated transition intensity. Thus this model seems to have accounted for a slow-transitioning mover population, instead of a stayer population.

In contrast, when *U_i_* is assumed inverse Gaussian, *g*(*u_i_*) → 0 when *u_i_* → 0 regardless of the parameter *ψ*. When *U_i_* is assumed CP distributed, *g*(*u_i_*) → *ρν* exp(−*ρ*) as *u_i_* → 0. It is then less likely that these distributions can place a large proportion of its mass arbitrarily close to zero. Thus, if a slow-transitioning mover population exists, the Poisson M-S inverse Gaussian and CP models may struggle to adequately represent these patients in the model for the movers, and therefore these models may attribute slow-transitioning movers with high stayer probabilities instead. Regarding the CP distribution, the parameter *ρ*, as discussed, governs both the overall baseline transition intensity, *ρ/ν*, and the stayer proportion, exp(−*ρ*). An overall slow transition intensity as indicated by *ρ* will then also enforce a higher stayer probability (through *ρ*) even if no stayer population exists. This is not the case for the other models, as a slow overall baseline transition intensity, (1 − *π*)λ_0_ through λ_0_—for example, when there are many slow transitioning movers—will not force *π* to take a certain value. These features may explain the greater estimated values of *π* from the Poisson M-S inverse Gaussian and CP models when compared with the Poisson M-S gamma model.

On balance, it thus seems sensible to regard the nonzero estimates of *π* to reflect either a stayer population or a subset of patients who are at minimal risk of the damage process as characterized by the specified distribution for the movers.

#### 11.1.2 Goodness of fit

The observed and estimated increments of damaged joints, for the various Poisson M-S models, can be compared in the following manner. Let

$$e_{ij}(y)=\hat{\text{P}}r(Y_{ij}=y\;|\;{\text{X}}_{ij,}=X_{ij},{\hat{\Lambda }}_{ij}),y=0,1,\ldots$$

be the estimated probability that the *i*th patient develops *y* additional damaged joints between *t_ij_* and *t*_*ij*+1_. For the Poisson M-S models, *Y_ij_* is assumed to have a Poisson distribution conditional on *u_i_*. In order to obtain values of *e_ij_*(*y*) for these models, the maximum likelihood estimates of Λ̂_*ij*_ and *π̂* are used along with empirical Bayes estimates of *u_i_* and the probability of being a mover for the *i*th patient (Yiu et al. 2016).

The observed and estimated changes in joint counts are displayed in columns 2–5 of Table 5. It is evident from the table that none of the Poisson M-S models provide particularly close agreements between the observed and estimated values. The category with increments of one damaged joint is considerably overestimated by all three models, which then results in the majority of categories with larger increments of damaged joints being severely underestimated. A statistic that accounts for the overall model performance at each category is the Pearson statistic. Let *o*(*y*) denote the observed number of times where *y* incremental damaged joints occurred, and let $e(y)=\sum_{i}\sum_{j}e_{ij}\left(y\right).$ A Pearson statistic can then be defined as $\sum_{y}\frac{{\left(0\left(y\right)-e\left(y\right)\right)}^{2}}{e\left(y\right)}.$ This statistic, with category >4 expanded to categories 4 to 8 and >8, was calculated as 221.49, 203.46 and 256.52 for the fitted Poisson M-S gamma, Poisson M-S inverse Gaussian, and Poisson M-S CP models, respectively. These numbers are relatively large and can be used for comparison purposes subsequently.

### 11.2 Negative Binomial Mover-Stayer Models

The lack of fit seen in the Poisson M-S models, as well as the discrepancies in the estimation of *π* between them, may suggest that the models should be extended to include observation-level random effects, as in Section 6.2. Yiu et al. (2016) reported the results of fitting these NB M-S models to the PsA data. The estimated values of *π* from the NB M-S inverse Gaussian and CP models were 0.3 (0.18, 0.45) and 0.34 (0.26, 0.43) respectively, and are therefore in much closer agreement than when observation-level random effects were not included. The profile likelihoods for all three models were very similar in shape to those from the Poisson M-S models, and again the profile log-likelihood for the NB M-S gamma model had its maximum at *π̂* = 0, with a 95% likelihood ratio interval (0, 0.086). Thus even after accounting for time-varying unobserved heterogeneity, the NB M-S gamma model provides no evidence for a stayer population, in contrast to the NB M-S inverse Gaussian and CP models. A (generalized) likelihood ratio test of *H*_0_ : *θ^nb^* = 0 resulted in *p* < 0.001 for each of the fitted NB M-S models, supporting the need to account for time-varying unobserved heterogeneity.

To compare observed and estimated incremental joint damage for these models, a similar method to that in Section 11.1.2 can be used (Yiu et al. 2016). Columns 6–8 of Table 5 display the estimated incremental joint damage from these NB M-S models. These models demonstrate a much improved fit to the data compared with the Poisson M-S models. As well, these models provide similar agreements between estimated and observed increments of damaged joints across all categories. In particular, there is some evidence that all three models still overestimate the category corresponding to an increase of one damaged joint. The Pearson goodness-of-fit statistics were calculated as 37.07, 38.06 and 35.89 for the fitted NB M-S Gamma, inverse Gaussian, and CP models, respectively, demonstrating much more reasonable agreements between the observed and estimated increments than for the models not incorporating observation-level heterogeneity. This is also demonstrated in the maximum log-likelihood values for the three estimated models, which are −2,249.9, −2,249.9 and −2,253.9, respectively, and which correspond to much greater likelihood values than those corresponding to the Poisson M-S models in Table 4.

As indicated in Section 6.2, a special case of the NB M-S Gamma and inverse Gaussian models is the ZINB model that can be viewed as a two-part model with observation level random effects, essentially a Poisson model with gamma random effects. This model leads to an estimate for *π* of 0.43 with a 95% CI of (0.36, 0.5). This model also results in a larger likelihood (log-likelihood = −2,279.36) compared with those in Table 4, but a likelihood ratio test of the hypothesis *θ* = 0, which corresponds to the ZINB model, within the NB M-S gamma model, is highly significant (test statistic = 29.5, *p*-value < 0.001). Thus, it appears that models with both patient- and observation-level random effects are the most appropriate for these PsA data.

## 12 Final Remarks

**Figure 6:**
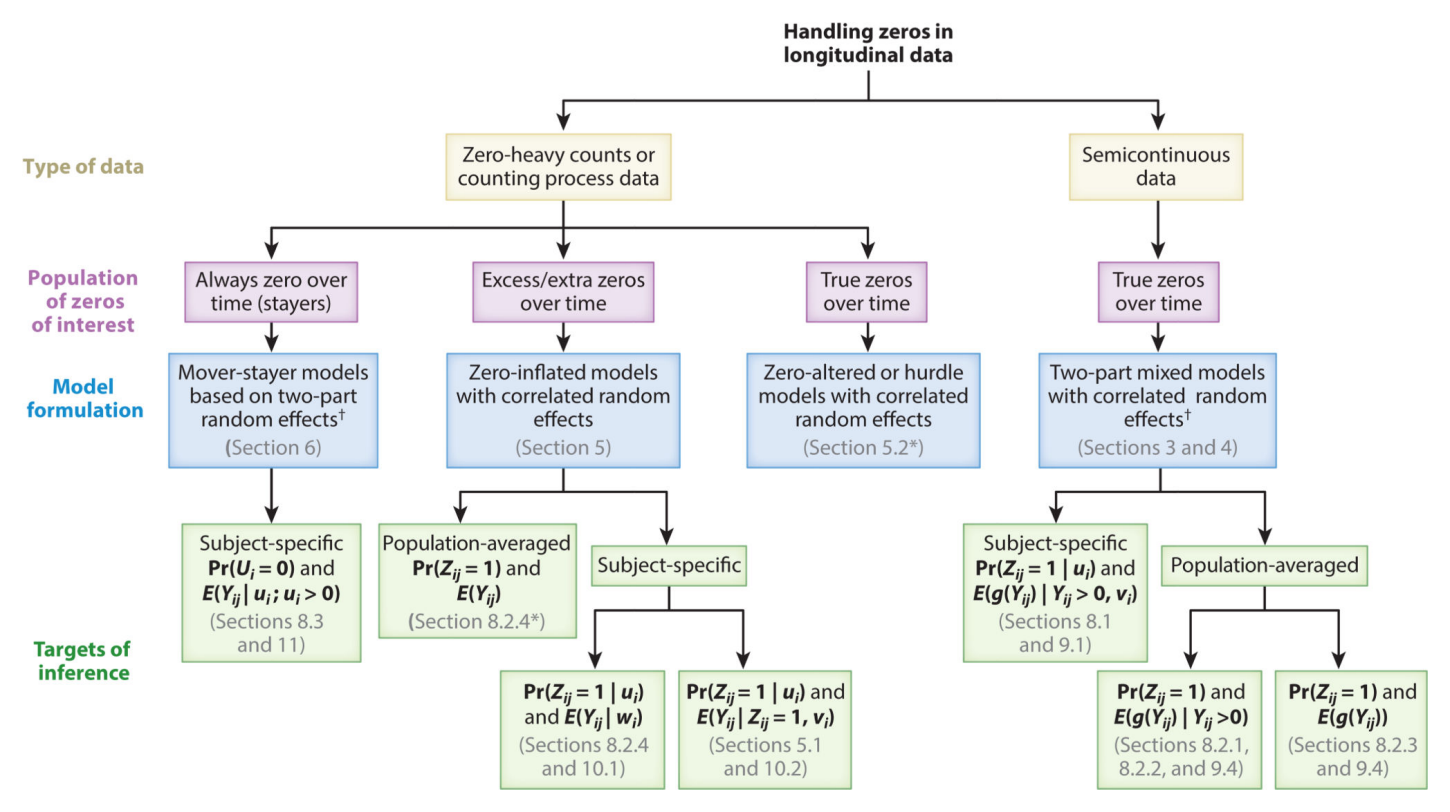
Schema of the different types of data, population of zeros of interest, model formulations and targets of inference discussed. An asterisk indicates that the topic is briefly discussed, and a cross indicates that different choices for the random effects distributions can be made.

We provide a survey of a variety of two-part models and consider issues arising in their use. Figure 6 provides a schema of the different data types, structures, models, and targets of inference discussed in this survey to aid the reader with the potential options, choices and considerations that can arise when dealing with longitudinal data of these types. Various models with a similar structure have been proposed, for example inflated beta regression models (Ospina & Ferrari 2012) and multistate models with a two-part structure (Young et al. 1999, O’Keeffe et al. 2012). The issues arising in the use of any such models for longitudinal data will be similar to those we have discussed.

There is scope, however, to consider models with slightly different structures. For example, as seen in Section 6.1.2, the CP distribution naturally contains a point mass at zero and a distribution along the positive real line, and therefore conveniently has a two-part structure. If this distribution is specified such that the mean of the summands and Poisson distribution is modeled using a log-link, then the overall mean will also be modeled using the log-link, with the linear predictor being the sum of the linear predictors from the component distributions. Thus, the CP distribution naturally provides easily interpretable inference on the overall mean and also maintains the intuitive structure of the means of the component distributions. Future investigation of this type of model would be of interest.

There is also scope for further development and use of methods to examine the goodness of fit of two-part models, perhaps especially for the representation of marginal covariate effects. In so doing, however, it is also important to recall that the adoption of models should also be based on the reasonableness of the particular two-part structure in the relevant scientific context, for example, when a not-at-risk or cured population of subjects is assumed. Thus, for example, models that account for an excess number of zeros without strong scientific assumptions may not be particularly useful.

In summary, this review considered two-part and related regression models in longitudinal settings where there are repeated measures over time from the same subject and there is a need to deal with zeros. We highlighted the need to assume correlation of random effects between the two parts exists rather than assuming independence, as the latter assumption will result in bias. We discussed different parameterizations and alternative forms of the mixed effects models that could be used in these settings, emphasizing that choices made should be dependent on the purpose, plausibility and particularities of the data. For example, the choice of parameterization used may depend on whether inference is to be made at a subject-specific or population-averaged level or whether the overall marginal mean, the conditional marginal mean, or the marginalized mean is the target of interest. Additionally, we discussed the possible sensitivity of estimation for, identifiability of, and inference on the stayer proportion to the chosen mover-stayer random effects distribution, especially when the random effects do not adequately capture the unexplained heterogeneity in the data.

## Figures and Tables

**Table 1:** Parameter estimates for the Health Assessment Questionnaire data

Parameters	Binary model		Continuous model	
	Misspecified estimate (SE)	Full estimate (SE)	Misspecified estimate (SE)	Full estimate (SE)
Intercept	−1.0199 (0.4079)	−1.0015 (0.3746)	0.3176 (0.0567)	0.2149 (0.0556)
Female	1.9944 (0.3603)	2.0080 (0.3276)	0.1811 (0.0505)	0.2225 (0.0512)
Disease duration	−0.0027 (0.0259)	0.0156 (0.0232)	0.0039 (0.0033)	0.0035 (0.0032)
AJ	0.1758 (0.0513)	0.1566 (0.0495)	0.0219 (0.0028)	0.0239 (0.0027)
DJ	−0.0161 (0.0321)	0.0120 (0.0260)	0.0058 (0.0031)	0.0052 (0.0031)
PASI score	0.1941 (0.1257)	0.1754 (0.1086)	0.0128 (0.0140)	0.0247 (0.0134)
AJ * duration	0.0002 (0.0034)	−0.0003 (0.0033)	−0.0004 (0.0002)	−0.0004 (0.0002)
DJ * duration	0.0032 (0.0016)	0.0022 (0.0013)	0.0002 (0.0001)	0.0003 (0.0001)
$\sigma_{u}^{2}$	4.2519 (0.8546)	4.3930 (0.8924)		
$\sigma_{v}^{2}$			0.1587 (0.0154)	0.1732 (0.0166)
$\sigma_{e}^{2}$			0.0785 (0.0040)	0.0774 (0.0039)
*ρ*	(*ρ* = 0)	0.9423 (0.0373)		
−2 log likelihood	2,116.0	2,018.1		
AIC	2,178.0	2,082.1		

Abbreviations and symbols: AIC, Akaike information criterion; AJ, active joints; DJ, deformed joints; PASI, Psoriasis Area and Severity Index; SE, standard error; $\sigma_{u}^{2}$, random effects variance for binary model; $\sigma_{v}^{2}$, random effects variance for continuous model; $\sigma_{e}^{2}$, random error variance; *ρ*, correlation between random effects in binary and continuous models.

**Table 2:** Parameter estimates in the binary and continuous parts from the two-part marginal model for the Health Assessment Questionnaire data: Marginal/conditional estimates in the binary part and the continuous part are in the form of log odds ratio and difference in means, respectively

	Binary part				Continuous part	
	Marginal estimate (SE)	*p*	Conditional estimate (SE*)	*p*	Conditional estimate (SE)	*p*
Intercept	0.62 (0.18)	0.0005	1.28 (0.37)	0.0005	0.46 (0.06)	<0.0001
*HLA-B27*	0.47 (0.22)	0.0324	0.97 (0.45)	0.0325	0.17 (0.08)	0.0294
*HLA-DQw3*	−0.22 (0.22)	0.3040	−0.46 (0.45)	0.3015	0.1075 (0.08)	0.16
*HLA-DR7*	−0.48 (0.29)	0.0972	−0.98 (0.59)	0.0964	−0.02 (0.10)	0.8775
HLA-DQw3*HLA-DR7	0.81 (0.38)	0.0358	1.66 (0.79)	0.0350	0.0256 (0.13)	0.85
Age at onset	0.40 (0.09)	<0.0001	0.82 (0.18)	<0.0001	0.10 (0.03)	0.0002
Disease duration	0.19 (0.07)	0.0072	0.39 (0.14)	0.0067	0.05 (0.02)	0.0182
Sex (female)	1.22 (0.19)	<0.0001	2.51 (0.41)	<0.0001	0.34 (0.06)	<0.0001
$\sigma_{b}^{2}$	10.64 (1.76)	<0.0001				
*ϕ*	0.49 (0.03)	<0.0001				
$\sigma_{v}^{2}$	0.29 (0.03)	<0.0001				
$\sigma_{e}^{2}$	0.09 (0.01)	<0.0001				
*ρ*	0.98 (0.02)	<0.0001				

* Obtained using the delta method.

Abbreviations and symbols: HLA, human leukocyte antigen (*B27, DQw3, DR7* are HLA alleles); SE, standard error; $\sigma_{b}^{2}$, random effects variance for binary model; *ϕ*, parameter of bridge density; $\sigma_{v}^{2}$, random effects variance for continuous model; $\sigma_{e}^{2}$, random error variance; *ρ*, correlation between pair of normal random variables used to construct random effects distributions.

**Table 3:** Marginalized zero-inflated Poisson model with random effects results: SafeTalk efficacy trial. Regression parameter estimates in the zero-inflation and marginalized mean models are in the form of log odds ratio and difference in log means, respectively

	Zero-inflation model			Marginalized mean model		
	Parameter	Parameter estimate	Robust SE	Parameter	Parameter estimate	Robust SE
Intercept	*θ*_0_	−2.1187	0.3665	*α*_0_	−0.8966	0.2965
Site 2	*θ*_1_	−0.1026	0.4184	*α*_1_	0.0362	0.2893
Site 3	*θ*_2_	−0.2445	0.9548	*α*_2_	−0.0220	0.6442
Follow-up 1	*θ*_3_	−1.2709	0.3468	*α*_3_	0.2011	0.1969
Follow-up 1* treatment	*θ*_4_	−0.8849	0.4627	*α*_4_	−1.2725	0.3365
Follow-up 2	*θ*_5_	−1.7071	0.7011	*α*_5_	−0.1217	0.2264
Follow-up 2* treatment	*θ*_6_	0.6021	0.9185	*α*_6_	−0.6128	0.3742
Follow-up 3	*θ*_7_	−1.0214	0.6881	*α*_7_	−0.4762	0.3521
Follow-up 3* treatment	*θ*_8_	0.3331	1.0968	*α*_8_	−0.2630	0.4691
Variance parameters†	*σ_u_*	9.7487	2.4313			
	*σ_uv_*	−4.5957	0.7345			
	*σ_v_*	3.4461	0.6599			

† $\hat{\rho }={\hat{\sigma }}_{uv}/(\sqrt{{\hat{\sigma }}_{u}{\hat{\sigma }}_{v}})=-0.79.$

Abbreviations and symbols: SE, standard error; *σ_u_*, random effects standard deviation for zero-inflation model; *σ_uv_*, square root of covariance of random effects from two parts of model; *σ_v_*, random effects standard deviation for marginalized mean model.

**Table 4:** Model fits for Poisson mover-stayer models. Estimated parameters and confidence intervals

	M-S Gamma	M-S IG	M-S CP
Previous number of damaged joints	–0.11 (–0.13, –0.10)	–0.12 (–0.14, –0.1)	–0.08 (–0.1, –0.07)
Previous number of active joints	0.06 (0.05, 0.07)	0.06 (0.05, 0.07)	0.06 (0.05, 0.08)
Arthritis duration at previous visit (years)	0.07 (0.06, 0.08)	0.07 (0.06, 0.09)	0.04 (0.03, 0.06)
Age at onset of arthritis	0.02 (0.005, 0.04)	0.02 (0.002, 0.04)	0.014 (0.003, 0.024)
λ_0_	0.03 (0.02, 0.07)	0.05 (0.02, 0.12)	1
*θ*	6.19 (5.11, 7.5)		
*ψ*		0.112 (0.066, 0.19)	
*ν*			12 (6.68, 17.32)
*ρ*			0.58 (0.49, 0.67)
ℙ(Stayer)	9.07×10^–4^ (5.5×10^–12^, 1)	0.32 (0.20, 0.47)	0.56 (0.51, 0.61)
Log-likelihood	–2,689.03	–2,676.34	–2,741.55

Abbreviations and symbols: CP, compound Poisson; IG, inverse Gaussian; M-S, mover-stayer; λ_0_, baseline mean rate parameter; *θ*, variance parameter of gamma distribution; *ψ*, reciprocal of variance parameter of inverse Gaussian distribution; *ν*, parameter of compound Poisson distribution; *ρ*, parameter of compound Poisson distribution.

**Table 5:** Observed and estimated changes in joint counts from the Poisson and negative binomial mover-stayer and zero-inflated models

Increments of damaged joints	Observed	P M-S gamma	P M-S IG	P M-S CP	NB M-S gamma	NB M-S IG	NB M-S CP
0 without previous damage	6,044	5,974.94	5,987.57	5,954.33	5,973.69	5,972.84	5,974.07
0 with previous damage	2,032	1,871.25	1,888.21	1,861.51	2,032.16	2,030.62	2,037.52
1	250	528.89	505.29	559.2	338.87	341.17	334.35
2	97	91.97	87.07	94.92	88.16	87.56	88.62
3	28	27.24	26.31	26.94	36.50	36.16	36.53
4	26	11.62	11.36	11.29	18.85	18.74	18.68
>4	53	24.09	24.19	21.81	41.78	42.89	40.24
Total	8,530	8,530	8,530	8,530	8,530	8,530	8,530

Abbreviations: CP, compound Poisson; IG, inverse Gaussian; M-S, mover-stayer; NB, negative binomial; P, Poisson.
